# Single-molecule live cell imaging of Rep reveals the dynamic interplay between an accessory replicative helicase and the replisome

**DOI:** 10.1093/nar/gkz298

**Published:** 2019-04-27

**Authors:** Aisha H Syeda, Adam J M Wollman, Alex L Hargreaves, Jamieson A L Howard, Jan-Gert Brüning, Peter McGlynn, Mark C Leake

**Affiliations:** 1 Department of Physics, University of York, York YO10 5DD, UK; 2 Department of Biology, University of York, York YO10 5DD, UK

## Abstract

DNA replication must cope with nucleoprotein barriers that impair efficient replisome translocation. Biochemical and genetic studies indicate accessory helicases play essential roles in replication in the presence of nucleoprotein barriers, but how they operate inside the cell is unclear. With high-speed single-molecule microscopy we observed genomically-encoded fluorescent constructs of the accessory helicase Rep and core replisome protein DnaQ in live *Escherichia coli* cells. We demonstrate that Rep colocalizes with 70% of replication forks, with a hexameric stoichiometry, indicating maximal occupancy of the single DnaB hexamer. Rep associates dynamically with the replisome with an average dwell time of 6.5 ms dependent on ATP hydrolysis, indicating rapid binding then translocation away from the fork. We also imaged PriC replication restart factor and observe Rep-replisome association is also dependent on PriC. Our findings suggest two Rep-replisome populations *in vivo*: one continually associating with DnaB then translocating away to aid nucleoprotein barrier removal ahead of the fork, another assisting PriC-dependent reloading of DnaB if replisome progression fails. These findings reveal how a single helicase at the replisome provides two independent ways of underpinning replication of protein-bound DNA, a problem all organisms face as they replicate their genomes.

## INTRODUCTION

Complex multienzyme systems produce high fidelity, complete copies of genomes prior to cell division but these replisomes face frequent barriers to their continued movement along DNA, threatening genome stability. Proteins bound to the template DNA are potential barriers, with the very high stability and abundance of transcribing RNA polymerases posing a particular challenge (1,2). Nucleoprotein barriers must be removed and replication resumed either by the original replisome or, if the blocked replisome dissociates, a replisome reloaded onto the DNA in a process known as replication restart (3). At the core of all replisomes are replicative helicases that unwind the template DNA and these replicative helicases may disrupt many, possibly most, of the potential nucleoprotein barriers encountered during genome duplication (4). However, the very high frequency of such collisions results in stochastic blockage of replisomes that requires additional mechanisms to ensure continued DNA replication (5,6). One such mechanism uses additional helicases to promote replisome movement along protein-bound DNA in bacteria and eukaryotes (5–10). However, since loading of the hexameric replicative helicase is tightly regulated to prevent over-replication (11), recruitment of other types of helicase therefore plays an important role in promoting replisome movement through nucleoprotein complexes.

All accessory replicative helicases identified to date are members of helicase Superfamily 1, members of which translocate as monomers along single-stranded DNA either in the 5′-3′ or in the 3′-5′ direction (12). Evidence is also emerging that at least one of these accessory helicases, Rep from *Escherichia coli*, has evolved features that optimise protein displacement from DNA (13). All accessory helicases studied so far have a polarity of translocation opposite that of the primary replicative helicase (4). Thus the *E. coli* accessory helicase Rep translocates 3′-5′ along single-stranded DNA (14) while the primary replicative helicase, DnaB, translocates 5′-3′ (15). Primary and accessory replicative helicases therefore translocate on opposing arms of the replication fork, which may allow additional motors to operate at a nucleoprotein block whilst the primary replicative helicase remains fully active at the fork (4). This arrangement may ensure that accessory helicases clear nucleoprotein barriers ahead of an active replisome that retains the primary replicative helicase, allowing resumption of replication by the same replisome without the dangers of blocked fork processing and replisome reloading (6,8,16–18).

Multiple monomers of Superfamily 1 helicases can function cooperatively to displace proteins from DNA (19). Having multiple accessory helicase monomers available at paused forks might therefore facilitate nucleoprotein complex removal (9). However, there is very little information concerning how accessory helicases interact physically and functionally with the replisome. The accessory helicases in *Saccharomyces cerevisiae* and *Schizosaccharomyces pombe*, Rrm3 and Pfh1 (5,20,21), interact with one or more subunits of the replisome (22–24). Similarly, *E. coli* Rep interacts via its C-terminus with the primary replicative helicase DnaB resulting in cooperative DNA unwinding and protein displacement by Rep and DnaB *in vitro* (6,9,25,26). There is the potential for up to six Rep monomers to associate with hexameric DnaB at the *E. coli* fork, supporting a model of multiple monomer recruitment to aid protein clearance (9). However, DnaB is a protein-protein interaction hub for the entire replisome (27) and so not all of the six DnaB subunits might be accessible to Rep. Indeed, a recent live cell single-molecule imaging study failed to detect any Rep molecules present at the replisome (28). Furthermore, accessory helicases at the fork may have more than one function and more than one interaction partner. Rep may interact functionally with the replication restart protein PriC to aid replisome reloading in the event of fork stalling and replisome dissociation (3,29,30). Rep may unwind the nascent lagging strand at such stalled forks to expose single-stranded DNA for PriC-directed loading of DnaB back onto the fork (30). Untangling the functions of Rep in promoting fork movement along protein-bound DNA and in replication restart is difficult, though. Loss of Rep accessory helicase function results in increased fork pausing and therefore fork breakdown, leading to an increased requirement for replication restart (31). How accessory replicative helicases operate within the context of replisomes to promote genome duplication remains obscure therefore.

Here, we use single-molecule microscopy of Rep in live *E. coli* cells and demonstrate that Rep colocalizes with ∼70% of replication forks. When present, there are six Rep monomers associated with each replisome, a stoichiometry that depends on the DnaB interaction motif within Rep, implying maximal occupancy of the single DnaB hexamer within the replisome. Rep molecules associate only transiently with the replisome, in part due to Rep-catalysed ATP hydrolysis, indicating dynamic association with the replisome and then translocation away from the fork. PriC is also involved in co-localization of Rep with the replisome, with loss of both the DnaB interaction motif within Rep and PriC being required to abolish colocalization of Rep with the replication fork. There are therefore two populations of Rep associated with replisomes *in vivo*. One population might involve Rep molecules continually associating with DnaB and then translocating away to aid nucleoprotein barrier removal ahead of the fork, while the second population might aid PriC-dependent reloading of DnaB in case replisome progression fails. These findings reveal for the first time the disposition of an accessory helicase within the context of a replication fork *in vivo*. They also reveal how a single type of helicase is recruited to the replisome to provide two ways of underpinning replication of protein-bound DNA, a problem that all organisms must face as they replicate their genomes.

## MATERIALS AND METHODS

### Cell strains

*Escherichia coli* clones comprising fluorescently tagged alleles of the *dnaQ, rep*, and *priC* genes (SI Table S2) were introduced into the respective native loci by lambda red recombineering (32), full details of growth curves (Supplementary Figure S1), cell doubling (SI Table S1), plasmids (SI Table S3), primers (SI Table S4) and methods are provided in *SI Text*. Cells were routinely grown overnight in LB at 37°C from freshly streaked LB plates. The LB grown cultures were then subcultured to mid-log phase at 30°C in 1 × 56 salts minimal medium with 0.2% glucose as the carbon source. The cells were then spotted onto slides overlaid with 1% agarose containing 1 × 56 salts and 0.2% glucose.

**Strain construction**

All strains used are derivatives of laboratory wild-type strain TB28. For tagging *dnaQ, linker-mGFPmut3* followed by a kanamycin resistance cassette flanked by *frt* sites was amplified by PCR from the plasmid pDHL580 (75) using primers oAS77 and oAS79, and *linker-mCherry-<kan>* was amplified from pJGB374 using primers oAS132 and oAS133 (SI Table S4). The amplification primers had a 50 bp homology at their 5′ end to the last 50 bp of the *dnaQ* gene preceding the stop codon (forward primer) or the 50 bp immediately after the stop codon (reverse primer). The resulting PCR products thus had homology either side such that recombination with the chromosome would result in in-frame integration of *linker-mGFP-<kan>* and *linker-mCherry-<kan>* immediately downstream of *dnaQ*, resulting in *dnaQ- mGFP-<kan>* and *dnaQ- mCherry-<kan>* alleles. PCR products were treated with *Dpn*I, gel purified, and introduced by electroporation into cells expressing the lambda Red genes from the plasmid pKD46 (32). Recombinants were selected for kanamycin resistance and screened for ampicillin sensitivity. The colonies obtained were verified for integration by PCR and sequencing with primers oAS84 and oAS85.

*mGFP-rep-<kan>* fusions for various *rep* alleles were amplified from plasmids pAS79 (*rep^+^*), pAS124 (*repC4ala*) and pAS127 (*rep2001*) with primers oAS141 and oJGB380 having 50 bp homology on either end of the native *rep* locus. Likewise *mCherry-rep-<kan>* was amplified from pJGB380 using primers oJGB379 and oJGB380. *mGFP-priC-<kan>* was amplified from the plasmid pAS65 using primers oAS136 and oJGB389. All PCR products were introduced on the chromosome of cells expressing lambda red genes at the native loci after *Dpn*I digestion, gel extraction, and electroporation as described above for *dnaQ* fusions. The *rep* recombinants were verified by PCR amplification and sequencing using the primers oJGB418, oMKG70, oMKG71, oPM363, oPM372 and oPM376. The *priC* recombinants were verified by PCR amplification and sequencing with primers oJGB402, oJGB403, oJGB417, and oJGB418. Where required, the kanamycin resistance gene was removed by expressing Flp recombinase from the plasmid pCP20 (32) to generate kanamycin sensitive strains carrying the FP fusions. Dual labelled strains were created by introducing the kanamycin tagged FP alleles by standard P1 mediated transduction into single labelled strains carrying the required FP allele after removing the linked kanamycin marker.

RepC4Ala

pBAD is a plasmid conferring kanamycin resistance that contains an arabinose-inducible promoter upstream of a multiple cloning site whilst pBAD*rep* is a derivative encoding wild type Rep (9). pBAD*repG672A,K673A* and pBAD*repK670A,R671A* were constructed by site-directed mutagenesis of the indicated codons within pBAD*rep*. pBAD*repC4Ala* is a derivative of pBAD*rep* in which all four codons were altered by site-directed mutagenesis to encode alanine. Assays to determine the ability of pBAD and derivatives to complement *Δrep ΔuvrD* inviability on rich medium were performed as described (9). Plasmid loss experiments to determine the viability of combinations of chromosomal alleles were performed as described (76).

### Microscopy and image analysis

A Slimfield microscope was used (33) for single-molecule imaging, an Olympus BX63 microscope measured epifluorescence. Foci tracking used MATLAB (Mathworks) software which determined *D* and stoichiometry using foci brightness (34) and Chung–Kennedy (35,69,70) filtered mYPet step-wise photobleaching and nearest-neighbour modelling (36).

A dual colour bespoke single-molecule microscope was used (33) which used a narrow 10μm at full width half maximum excitation field at the sample plane to generate Slimfield illumination. Excitation was from 488 and 561 nm, 50 mW Obis lasers digitally modulated to produce alternating laser excitation with 5 ms period. Modulation was produced by National Instruments dynamic I/O module NI 9402. Excitation was coupled into a Zeiss microscope body with a Mad City Lab's nanostage holding the sample. Emission was magnified to 80 nm/pixel and imaged using an Andor Ixon 128 emCCD camera. Green/Red images were split using a bespoke colour splitter consisting of a dual-pass green/red dichroic mirror centred at long-pass wavelength 560 nm and emission filters with 25 nm bandwidths centred at 542 and 594 nm. Samples were imaged on agarose pads suffused with media as described previously (72).

Foci were automatically detected and tracked using bespoke MATLAB software described previously (34). In brief, bright foci were identified by image transformation and thresholding. The centroid of candidate foci were determined using iterative Gaussian masking (77) and accepted if their intensity was greater than a signal to noise ratio (SNR) of 0.4. Intensity was defined as the summed pixel intensity inside a 5 pixel circular region of interest (ROI) corrected for the background in an outer square ROI of 17 × 17 pixels. SNR was defined as the mean BG corrected pixel intensity in the circular ROI divided by the standard deviation in the square ROI. Foci were linked together into trajectories between frames if they were within 5 pixels of each other. Linked foci were accepted as ‘tracks’ nominally if they persist for at least four consecutive image frames, unless specified otherwise.

Stoichiometry was determined by fitting the first three intensity values of a foci to a straight line, using the intercept as the initial intensity and dividing this by the characteristic intensity of GFP or mCherry. This characteristic intensity was determined from the distribution of foci intensity values towards the end of the photobleach confirmed by overtracking foci beyond their bleaching to generate individual photobleach steps of the characteristic intensity (Supplementary figure S2). The number of peaks in the Gaussian fits to Rep was set by running a peak fitting algorithm over the wild type distribution. This number of Gaussians was then used for mutant distributions unless two or more of the Gaussians converged on the same/similar peak value, in which case they were removed. For DnaQ, two peaks were fit as used previously (40).

Red and green images were aligned based on the peak of the 2D cross correlation between brightfield images. Colocalization between foci and the probability of random colocalization was determined as described previously (36). Microscopic diffusion coefficients were calculated by fitting the first three mean square displacement (MSD) values, i.e. equivalent to time interval values of 5, 10 and 15 ms, with a linear fit constrained through the equivalent localization precision MSD (78). Dwell time was calculated as the number of frames that each trajectory was colocalized with the fork position, as determined by the DnaQ foci detected at time zero.

### Dual labelled Rep/DnaB

Preliminary attempts to construct a DnaB-mCherry fusion resulted in non-viable filamentous cells. However, we managed to construct a viable non-filamentous strain using an existing strain which contained a mYPet-DnaB fusion (64) into which we then moved the mCherry-Rep fusion. This resulted in resolvable DnaB-mYPet and Rep-mCherry foci, albeit with less optimal photophysical properties compared to mGFP/mCherry imaging due to the higher peak emission wavelength of mYPet compared to mGFP and relative dimness and photo-instability of mCherry compared to mYPet, but still indicating similar numbers of foci per cell as measured for the DnaQ replication fork marker in our other Rep/DnaQ strains. Applying criteria such that foci were accepted with only two consecutive image frames compared to the default of four to account for more rapid photobleaching of mCherry compared to mYPet, resulted in >200 mCherry-Rep foci across N = 77 cells, with 45 ± 5% of these colocalized to mYpet-DnaB (note, using the default foci detection criteria resulted in only 11 Rep-mCherry foci tracks detected from these 77 cells, compared to >200 DnaB-mYPet foci, however, of these the proportion that were colocalized with mYpet-DnaB foci was still measured as ∼45%).

### Overexpression and Purification of mGFP-Rep and mGFP-RepC4ala

*mGFP-rep* and *mGFP-repC4ala* were sub-cloned from pAS79 and pAS124 respectively using XhoI and BamHI before ligation into pET14b cut similarly, creating pJLH237 and pJLH238 encoding histidine-tagged mGFP-Rep and histidine-tagged mGFP-RepC4ala respectively. pJLH237 and 238 were used to overexpress the mGFP-Rep fusions in HB222. Growth was carried out in F-Medium (79) at 37°C until OD_600_ ∼0.7, overexpression was induced by the addition of 0.2% arabinose (w/v) and 1 mM IPTG for 3 h at 20°C. Cells were pelleted by centrifugation at 5000 × g for 20 min at 4°C before flash freezing in 50 mM Tris–HCl pH 7.5, 10% sucrose (w/v) and storage at –80°C. Cell pellets were thawed on ice and the following additions were made (final concentrations indicated) 50 mM Tris–Cl pH 8.4, 20 mM EDTA pH 8.0, 150 mM KCl and 0.2 mg ml^−1^ lysozyme. After 10 min incubation on ice, Brij-58 was added to 0.1% (v/v of final concentration) with a further 20 min incubation on ice. The mixture was clarified by centrifugation at 148 000 × g for 1 h at 4°C and the supernatant recovered. DNA was precipitated from the resultant supernatant by dropwise addition of Polymin P to 0.075% (v/v) with stirring at 4°C for 10 min. The supernatant was recovered by centrifugation (30 000 × g, 4°C for 20 min) before solid ammonium sulfate was added to 50% saturation whilst stirring at 4°C for 10 min. The pellet was recovered by centrifugation at 30 000 × g at 4°C for 20 min and stored on ice overnight at 4°C. The protein pellet was then diluted in 20 mM Tris–HCl pH 7.9 and 5 mM imidazole until the conductivity matched that of 20 mM Tris–HCl pH 7.9 and 500 mM NaCl (buffer A) plus 5 mM imidazole. The Rep fusion proteins were purified by chromatography on a 1 ml His-trap FF crude column (GE healthcare) using a 20 ml wash with buffer A + 20 mM imidazole and a 20 ml gradient 20 mM to 1 M imidazole in buffer A, collecting 0.25 ml fractions. Peak fractions (∼120 mM imidazole) were collected, and a Vivaspin 20 concentrator (100 kDa MWCO) (Sartorius) was used to assess for concentration levels and for buffer exchange into 20 mM Tris–HCl pH 8.0, 500 mM NaCl, 1 mM EDTA, 1 mM DTT, 30% glycerol (v/v). Samples were then aliquoted and flash frozen in liquid nitrogen before storage at –80°C. Protein concentrations were determined by Bradford's assay.

### Helicase assay

Unwinding of streptavidin-bound forks was assayed using a substrate made by annealing oligonucleotides oPM187B20 and oPM188B34. Reactions were performed in final volumes of 10 μl in 50 mM HEPES (pH 8); 10 mM DTT; 10 mM magnesium acetate; 2 mM ATP; 0.1 mg ml^−1^ BSA and 1 nM forked DNA substrate. Briefly, the reaction mixture was pre-incubated at 37°C for five minutes ±1 μM streptavidin (Sigma-Aldrich), then histidine-tagged helicase (as indicated) and biotin (Sigma-Aldrich) to 100 μM (acting as a trap for free Streptavidin) were added and incubation continued at 37°C for 10 min. Reactions were stopped with 2.5 μl of 2.5% SDS, 200 mM EDTA and 10 mg ml^−1^ of proteinase K. Reactions were then analysed by non-denaturing gel electrophoresis on 10% polyacrylamide TBE gels.

Expanded details can be found in *SI Text*.

## RESULTS

### Rep hexamers associate with most replication forks, with monomeric Rep diffuse in the cytoplasm

We set out to test the extent of association between Rep and functional replication forks, and what mediates this interaction. To report on the replisome position we replaced the wild type *dnaQ* gene on the chromosome with a C-terminal *dnaQ-mCherry* fusion construct (see SI Text) using lambda red recombineering (32) as well as replacing either wild type copies of *rep* or *priC* genes with N-terminal monomeric GFP (mGFP) fusions (37) *mGFP-rep* and *mGFP-priC* respectively to generate two dual-label strains expressing either mGFP-Rep or mGFP-PriC (preliminary experiments indicated that the N-terminal fusions were closer to the WT phenotype than the C-terminal fusions), with a DnaQ-mCherry fork marker, both with wild type levels of functional activity (Supplementary Figure S2; SI Table S1). To observe the dynamic patterns of Rep and PriC localization in the cell relative to the replication fork we used single-molecule Slimfield imaging (38). This optical microscopic technique allows detection of fluorescently-labelled proteins with millisecond sampling to within 40 nm precision (39), enabling real time quantification of stoichiometry and mobility of tracked molecular complexes inside living cells, exploited previously to study functional proteins involved in DNA replication and remodelling in bacteria (40,41), bacterial cell division (42), eukaryotic gene regulation (33), and chemokine signalling in lymph nodes (43).

**Figure 1.**
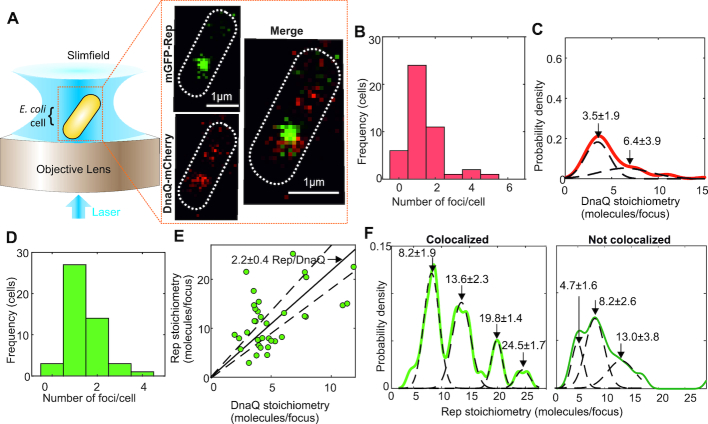
Single-molecule Slimfield of DnaQ-mCherry and mGFP-Rep. (**A**) Slimfield schematic and example images of mGFP-Rep (green) and DnaQ-mCherry (red). (**B**) Histogram showing DnaQ-mCherry foci detected per cell. (**C**) Kernel density estimate of number of DnaQ-mCherry molecules per focus with two Gaussian fit, means and SEM indicated. (**D**) Number of Rep foci per cell. (**E**) Rep versus DnaQ stoichiometry for all colocalized foci (green), linear fit (black line) constrained through origin indicated with gradient ±95% confidence interval on the gradient (dotted lines). Note the equivalent 1 SD error for this fit is 0.2 Rep molecules per DnaQ, and so the probability that the nearest integer ratio is 2 Rep molecules per DnaQ is very high. (**F**) Stoichiometry of Rep foci colocalized and not to DnaQ, multiple Gaussian fits shown with mean ± SD indicated. *N* = 45 cells.

We grew cells to mid-logarithmic phase then immobilized cells onto agarose pads suffused with growth medium for imaging. Slimfield indicated mostly one or two replication forks per cell (Figure 1A,B), which manifested as distinct fluorescent foci of diffraction-limited width ∼300 nm, as expected for cells undergoing mainly one round of chromosomal duplication per cell cycle as we have in our growth conditions (40). Using step-wise photobleaching analysis of the mCherry tag we could accurately quantify the stoichiometry of these foci (Supplementary Figure S3) indicating peaks centered on three or six DnaQ molecules per focus (Figure 1C) with a small minority having greater than six DnaQ per focus to be compared with previous observations from live cell fluorescence microscopy (40,44) indicating three DNA polymerases per replisome (45,46), or six per focus when two replication forks are sufficiently close so that they cannot be resolved optically, or more rarely greater than six for some cells starting a second round of replication. Replacing the fluorophore with mGFP (Supplementary Figure S4) yielded similar stoichiometries but with more foci detected per cell consistent with its smaller point spread function width and higher emission signal relative to mCherry (41).

In the same DnaQ-mCherry containing cells we observed mostly one or two mGFP-Rep foci per cell (Figure 1D). By computing the numerical overlap integral between foci in the red and green detection channels (36) we could robustly determine the extent of colocalization between Rep and DnaQ to within 40 nm localization precision. These analyses indicated that 70±7% (±SE, *N* = 70 foci) of Rep foci were colocalized with DnaQ, with both the colocalized and non-colocalized populations having similar ranges of stoichiometry equivalent to ∼6–30 Rep molecules per focus, however, only colocalized Rep displayed distinct periodicity in stoichiometry. Rep stoichiometry was correlated to DnaQ stoichiometry (correlation coefficient, *R* = 0.48). A linear fit of Rep to DnaQ stoichiometry, forced through the origin, resulted in a poor fit (*R*^2^ ∼ 0) but showed approximately two Rep molecules associated per DnaQ molecule (Figure 1E, F), within 95% confidence error threshold. Since each replisome contains an average of three DnaQ molecules (40,45) our data indicate there are an average of six Rep molecules present at each replisome (the mean separation of all of the Gaussian peaks shown in Figure 1F is 5.5 ± 0.8 Rep molecules, or 6 molecules to the nearest integer), consistent with our measurement of the periodicity of Rep stoichiometry colocalized with DnaQ (Figure 1F). However, the absolute values of the stoichiometry peaks (integer values of 8, 14, 20, 25 Rep molecules) are marginally higher by ∼1–2 molecules compared to what we might nominally expect for hexameric Rep (i.e. 6, 12, 18, 24 molecule peaks) due to detection of diffusive mGFP-Rep molecules in the cytoplasmic pool in addition to the hexameric Rep at the relatively high copy numbers we measure here (see SI Text).

An average of six Rep molecules associated with each replisome implies full occupancy of Rep binding sites on the DnaB hexamer within each replisome (9). Although we cannot exclude that a proportion of Rep is associated with the DNA directly and not DnaB, the observed hexameric periodicity of colocalized Rep (Figure 1F) adds more support to a model in which Rep interacts directly with DnaB. We confirmed direct association of Rep and DnaB by constructing and imaging a dual-label DnaB-mYPet:Rep-mCherry strain, indicating that 45 ± 5% of detected Rep-mCherry foci were colocalized with DnaB-mYPet foci (Supplementary Figure S5 and SI Text).

As well as distinct foci, as alluded to above we also detected a diffuse pool of Rep fluorescence throughout the cell, similar to previous studies of *E. coli* replisome proteins (40). Using numerical integration of cellular pixel intensities (47) we quantified the pool copy number to be several hundred Rep molecules per cell (Supplementary Figure S6) comparable to that estimated previously using quantitative western blots on cell lysates (48). We can estimate the stoichiometry of Rep foci in the pool using nearest neighbour analysis (33), since by definition pool foci must be separated by less than the optical resolution limit of our microscope which is ∼230 nm, indicating monomeric Rep in the pool (see SI Text).

### Rep-fork association is mediated by the DnaB interaction motif within Rep

The Rep–DnaB interaction resides within the C-terminal 33 amino acids of Rep and consequently the *rep*Δ*C33* allele displays a partial loss of *rep* function (9,48). To test whether the patterns of colocalization between Rep and the replisome we observed were due to the Rep–DnaB interaction we constructed an *mGFP-repΔC33* fusion (activity data summarized in Supplementary Figure S7; Supplementary Table S1). However, the fusion had a negative impact on *rep*Δ*C33* function (see Supplementary Figure S7C comparing iii with iv). We therefore searched for mutations within the C-terminal 33 codons that would recapitulate the *rep*Δ*C33* phenotype but would otherwise retain function when fused to *mGFP*. We found that mutating the final four codons of *rep* encoding KRGK to encode alanine resulted in an allele displaying a partial loss of function similar to *rep*Δ*C33* but which could be fused to *mGFP* without a complete loss of function.

Both mGFP-Rep and mGFP-RepC4Ala are functional *in vitro* and possess a higher level of activity than the wild type protein (Supplementary Figure S8) (13,26). Whilst this increase in activity could be attributed to the mGFP fusion causing oligomerization of the protein and a shift to a more active state (49–51) *in vitro*, the free pool of mGFP-Rep appears monomeric *in vivo*. We also believe mGFP is unlikely to be causing oligomerisation given the K_d_ for dimerisation of mGFP has been measured as 74 mM (37), and previous work has shown mGFP to have no effect on the oligomeric state of other fusion proteins (33). An alternative hypothesis is that the mGFP fusion is aiding the solubility of Rep *in vitro* (52) similarly to other large fusion tags such as MBP (53,54). We therefore believe it is likely that the partial loss of function displayed by the *repC4ala* allele *in vivo* is due to the inability of RepC4Ala to interact with DnaB rather than any inherent loss of function of the mutant protein.

**Figure 2.**
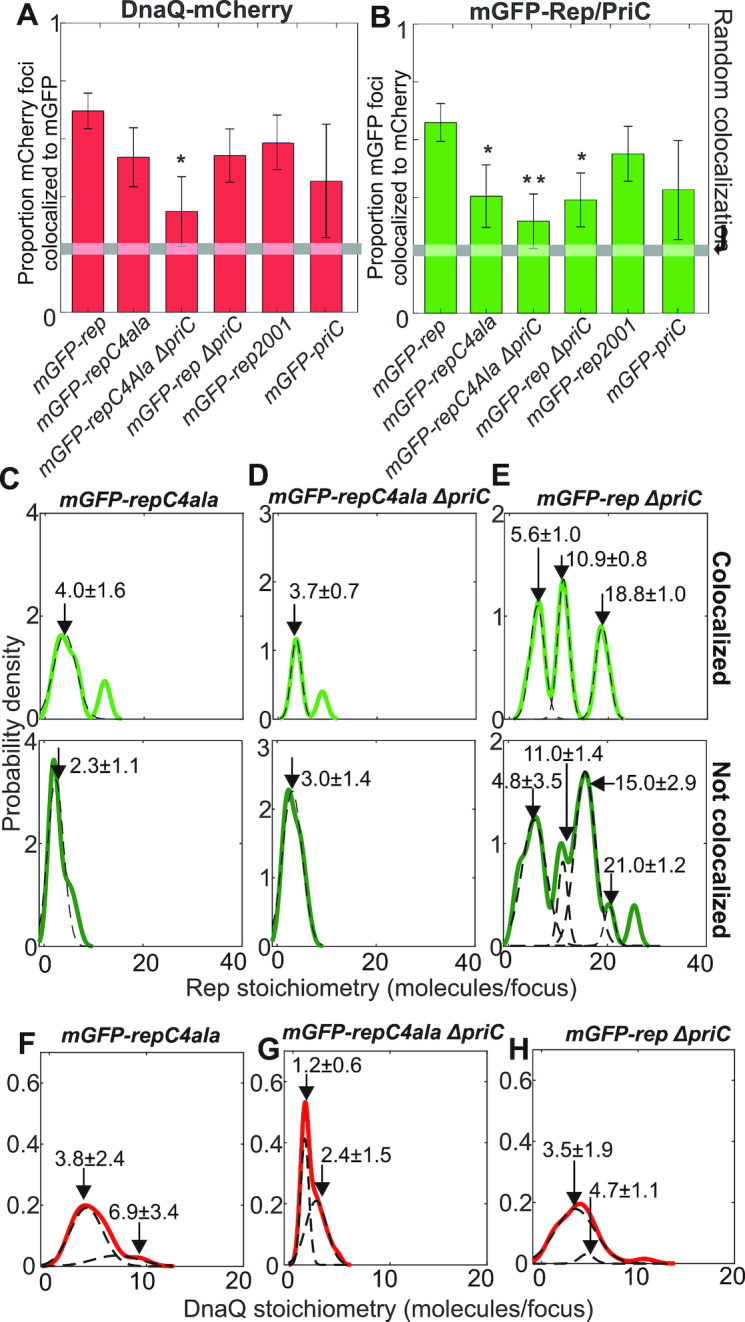
Rep/DnaQ colocalization analysis. Proportions of (**A**) DnaQ-mCherry foci colocalized with mGFP-Rep or mGFP-PriC, (**B**) mGFP-Rep or mGFP-PriC foci colocalized with DnaQ-mCherry. All strains carry *dnaQ-mCherry* allele with relevant genotypes indicated, gray horizontal bar indicates random colocalization (i.e. foci overlap) level based on our simulations, significance at *P* < 0.05 (*) indicated. The mGFP-repC4ala and the mGFP-*rep ΔpriC* are both significant at *P* < 0.05 (*) and the double mutant is significant at *P* < 0.01 (**), *P* = 0.048, 0.027 and 0.006 respectively from left to right. (**C–E**) Kernel density estimates of number of mGFP-Rep molecules in foci colocalized with DnaQ-mCherry (light green) and foci not colocalized with DnaQ-mCherry (dark green) in wild type and mutant backgrounds. Multiple Gaussian fits (dotted lines) and mean values ± SD indicated. (**F–H**) Kernel density estimates of the number of DnaQ-mCherry in each focus in wild type and mutant backgrounds, multiple Gaussian fits (dotted lines) and mean values ± SD indicated. *N* = 30 cells.

When *mGFP-repC4ala* was introduced into the *dnaQ-mCherry* strain, the fraction of colocalized Rep–DnaQ foci dropped significantly (Figure 2A and B) but similar numbers of foci were detected (Supplementary Figure S9). The stoichiometry of RepC4Ala foci dropped to 2–4 molecules per focus independently of their position relative to the fork, however, we observed that fork-colocalized RepC4Ala foci lost the pattern of periodicity in the stoichiometry distribution that we observed with mGFP-Rep (compare Figure 2C with 1F); this observation suggests a key role for the Rep C terminus in specifying its putative hexameric stoichiometry when in the vicinity of the replication fork. However, levels of colocalization seen with RepC4Ala were still above those expected for purely random optical overlap of Rep and DnaQ foci (Figure 2A and B). This non-random association indicates either that RepC4Ala can still interact with DnaB to some extent or that Rep can associate with the replisome independent of the Rep–DnaB interaction. The significant decrease in the number of RepC4Ala molecules within foci that are not colocalized with DnaQ as compared with wild type Rep (compare Figure 2C with Figure 1F) also indicate that the Rep C-terminus plays a role in the formation of Rep oligomers in the absence of any direct association with the replication fork.

### Association of Rep and replication forks is modulated by PriC

Biochemical and genetic evidence indicates that Rep also participates in PriC-dependent fork reloading (29,30). However, evidence of a physical association between PriC and Rep is lacking, prompting us to employ functional imaging of PriC in live cells. We used an *mGFP-priC* fusion that retained wild type function. Although the cell doubling time for this strain was higher than wild type (see Supplementary Table S1), indicating some level of fitness cost, our tests using a plasmid loss assay *in vivo* (Supplementary Figure S2B) indicated that the fusion construct is functional. We found that ∼40% of DnaQ foci contained PriC (Figure 2A, Supplementary Figure S10). We did not characterize this protein *in vitro* because this strain was only used to demonstrate that PriC was localized at the fork *in vivo*. Thus a significant minority of replisomes contain PriC.

The impact of PriC on the colocalization of DnaQ and Rep was probed by deleting *priC*. A *ΔpriC* mutation reduced the proportion of Rep–DnaQ colocalized foci (Figure 2B). The probability of Rep association with the replisome is therefore determined in part by PriC. In contrast, the range of stoichiometries of Rep molecules in foci colocalized with DnaQ was relatively unaffected when comparing *priC^+^* and Δ*priC* strains, and the hexameric periodicity in stoichiometry remained (compare Figures 1F and 2E), which contrasts with the marked impact of the *repC4ala* mutation. These data indicate that the pronounced periodicity in the patterns of association of Rep with the replisome is dependent on the Rep C-terminus rather than PriC.

Combining both *repC4ala* and *ΔpriC* mutations reduced the incidence of RepC4Ala colocalization with DnaQ to levels consistent with random association with the replisome (Figure 2A and B). Thus both the Rep C-terminus and PriC contribute to association of Rep with the replisome. However, the stoichiometry of RepC4Ala foci associated with DnaQ in the *repC4ala ΔpriC* double mutant strain was similar to the single *repC4ala* mutant (Figure 2, compare C and D). The significant periodicity in patterns of association of Rep with the replisome is determined therefore by the Rep C-terminus rather than PriC. Replisome composition was also affected in the *repC4ala ΔpriC* double mutant since the number of DnaQ molecules was reduced from 3–6 to 1–2 molecules per focus (compare Figure 2G with 1C).

Deleting *priC* also altered the pattern of Rep stoichiometry in foci not colocalized with the replisome (compare Figure 1F with Figure 2E). However, there were still significant numbers of Rep molecules in foci far from the replisome in *ΔpriC* cells which was in marked contrast to the major reduction in numbers of RepC4Ala molecules in foci far from the replisome in *priC^+^* cells (compare Figure 2C and E). These data indicate that the Rep C-terminus is the primary determinant of Rep oligomer formation far from the replisome, as with focus formation at the replisome.

### Rep-fork interactions are transient, dynamic and ATP dependent

The generally accepted model of Rep accessory helicase function is that Rep associated with the replisome translocates along the single-stranded leading strand template and unwinds the parental dsDNA, whilst simultaneously promoting dissociation of any proteins bound to this dsDNA (9) (see also Figure 4). Rep might therefore translocate in an ATP-dependent manner away from the replisome in addition to any spontaneous dissociation. We probed therefore the ATP dependence of Rep–DnaQ dissociation, and its dynamics.

**Figure 3.**
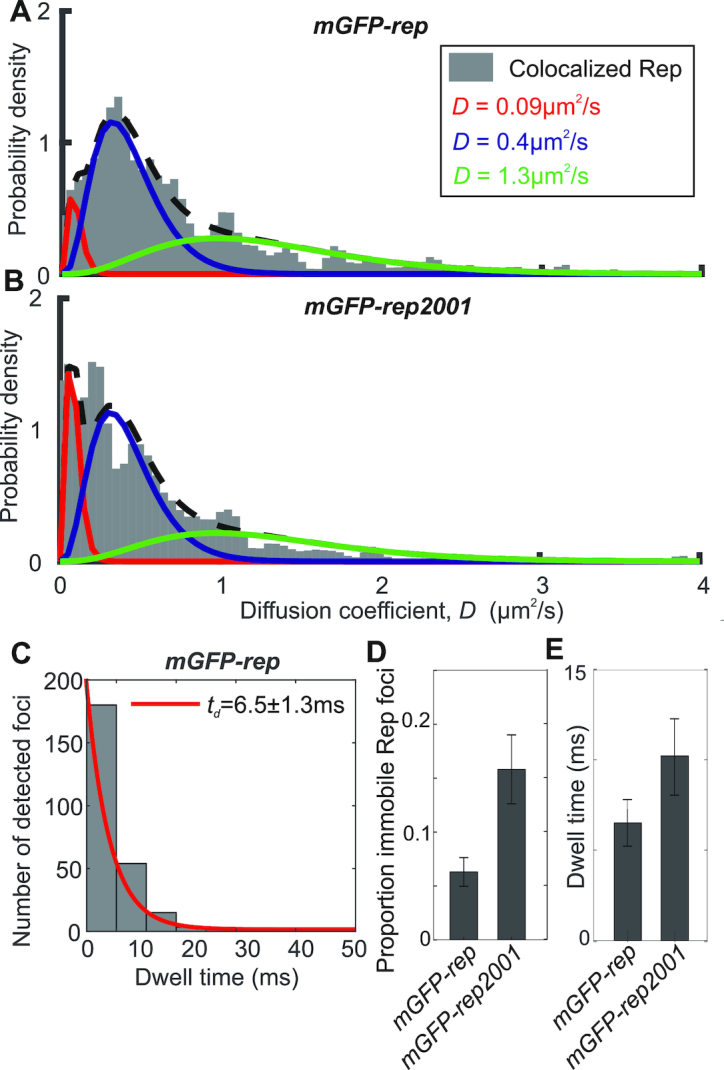
Rep mobility analysis. (**A** and **B**) Binned kernel density estimates (grey) of mGFP-Rep and mGFP-RepK28R diffusion coefficient distributions with three Gamma curve diffusion coefficient fits, minimal reduced χ^2^ = 0.0067, proportion in each model indicated. (**C**) mGFP-Rep foci dwell time with mCherry-DnaQ foci distribution with an exponential fit (red). (**D**) Proportion of colocalized Rep foci that are immobile, as determined from the three Gamma curve fits. (**E**) Histogram for the distribution of mean dwell time derived from fits for mGFP-Rep and mGFP-RepK28R. Error bars are 95% confidence intervals. *N* = 45 cells for wild type and 30 per mutant, with ∼300 trajectories.

Rep foci appeared highly dynamic (Supplementary Movie 1 and Movie 2). We analysed their mobility on the millisecond timescale, correlated to their state of localization with the fork, by calculating the microscopic diffusion coefficient *D* of each tracked focus and fitting a model consisting of the sum of multiple gamma functions model (43). A three parameter model fitted the data best (Figure 3A and B, Supplementary Figure S11 and SI Table S5) comprising *D* = 0.09 μm^2^/s, consistent with immobile foci based on our tracking localization precision of 40 nm, in addition to a slow (mean *D* = 0.4 μm^2^/s) and a fast (mean *D* = 1.3 μm^2^/s) diffusion mode. The immobile state is consistent with Rep binding to the fork. Fast diffusion is broadly consistent with expectations of free diffusion in the cytoplasm considering estimates of the likely hydrodynamic drag radius of the mGFP-Rep construct: for example, assuming a mean cytoplasmic viscosity of ∼10 cP (55) then the very fastest diffusion that we can track occurs at values of ∼4 μm^2^/s indicates an approximate hydrodynamic drag radius of ∼5 nm. Similar ‘slow diffusion’ was recently observed for other DNA repair proteins – UvrA and B (56), as well as for DNA gyrase (57), and attributed to transient protein binding to DNA.

To probe the dependence on ATP hydrolysis we labelled a mutant RepK28R, encoded by the *rep2001* allele (25), with mGFP, whose mutation lies in the Walker A domain that is essential for ATP hydrolysis and hence translocation along DNA (48,58). The mGFP-RepK28R fusion retained the ability to associate with DnaQ, as evidenced by a similar proportion of colocalized mGFP-RepK28R and DnaQ foci as compared with mGFP-Rep (Figure 2B). Also, the distributions of RepK28R stoichiometry (Supplementary Figure S9) and total cell copy number (Supplementary Figure S6) were similar to wild type. However, mGFP-RepK28R also showed a significant increase in the proportion of immobile colocalized foci from 6 ± 1% in the wild type to 15±3% (compare Figure 3B with A; Figure 3D; Supplementary Figure S11). This increase contrasted with Rep foci not colocalized with the fork, which failed to show a significant difference between wild type and RepK28R (Supplementary Figure S11C).

We estimated the dwell time of Rep foci at the replication fork from the number of consecutive image frames associated with each colocalized track. The distribution of dwell times decreased exponentially with a characteristic time constant of 6.5 ± 1.3 ms at the fork for wild type Rep, increasing to 10.2 ± 2.1 ms with RepK28R (Figure 3C and E, and Supplementary Figure S11B). Dwell time fits to the *repC4ala* mutation and *priC* deletion based on a single exponential model were poor, suggesting that there are likely to be a range of factors influencing dwell time: for example, the kinetics of binding to and unbinding from single-stranded DNA, and the frequency with which single-stranded DNA regions become available and accessible, which we propose to investigate in future studies. We conclude that when Rep is able to hydrolyze ATP, a smaller proportion of Rep molecules are immobile at the replisome and these immobile molecules also spend significantly less time at the fork. These data imply that dissociation of Rep from the replisome is driven in part by ATP-dependent translocation of Rep along DNA.

## DISCUSSION

Here, we show that the majority of replisomes contain the accessory replicative helicase Rep, that there are approximately six Rep molecules per replisome and that this distribution is dependent upon the Rep C-terminus (Figures 1 and 2). The only known function of the Rep C-terminus is to interact physically with DnaB (9,25). These data are consistent therefore with Rep association being driven primarily by the Rep–DnaB interaction (9) and suggest high occupancy of the six potential Rep binding sites within the DnaB hexamer at the replisome. Our data also demonstrate rapid turnover of Rep at the replisome and the importance of Rep-catalyzed ATP hydrolysis for this rapid turnover (Figure 3). These findings suggest a model in which the majority of replisomes have near-full occupancy of Rep binding sites and that these Rep molecules bind continually to single-stranded DNA at the fork to translocate ahead of the advancing replisome to help displace proteins from the template. We also find that association of Rep with the replisome is dependent in part on PriC (29,30) (Figure 2B), consistent with the functional interaction between Rep and PriC in replication restart. Whether this PriC-dependent association of Rep with the replisome is due to a direct Rep–PriC interaction or due to an indirect effect of PriC is unknown. These data do indicate, though, that there may be a complex interplay between DnaB and PriC in terms of Rep function within the replisome.

Our data also demonstrate that a minority of Rep foci form away from any replisomes (Figures 1F and 2B) with the number of Rep molecules within these foci again dependent primarily on the DnaB interaction motif within the Rep C-terminus (compare Figure 1F with Figure 2C). DnaB hexamers can be loaded onto single-stranded DNA only with the aid of the helicase loader DnaC (59–62) implying that at least some of the DnaB not associated with replisomes is bound by DnaC in a DnaB_6_:DnaC_6_ complex (63). Our data indicate that at least some of this DnaB not within replisomes is associated with Rep, consistent with earlier observations for live cell fluorescence microscopy that mobile DnaB foci can be detected diffusing away from replication forks in addition to an immobile replisome-anchoring population (64). The binding of Rep and DnaC to DnaB appears to be mutually exclusive (9) implying that Rep and DnaC are in competition for binding of the pool of DnaB away from replisomes.

Our finding of multiple Rep molecules colocalized with the replisome compares to a recent live cell imaging study of fluorescently-labelled Rep, and other repair and replisome proteins (28). Here, although the authors did not have an independent fork marker for visualizing simultaneous Rep and fork colocalization, they observed Rep foci in locations consistent with fork localization. They reported populations of Rep foci which were relatively stable in appearing in at least four consecutive image frames, but also a significant number of foci that lasted for fewer than four consecutive frames. The total proportion of cells exhibiting detectable Rep foci was ∼70% (comprising 32% stable and 38% unstable foci in reference to the relative transience of their appearance on consecutive image frames as defined by the authors), similar to our observations here for the proportion of all detected Rep foci which are colocalized with the replication fork marker. Our observations are consistent with these previous findings in light of the very rapid dynamics of Rep we measure (average dwell time of ∼6 ms at the fork) which is significantly faster than the earlier study could sample with a frame integration time of 40 ms strobed every 200 ms. Coupled to this Rep foci detection in this earlier study was also limited to a reported sensitivity of at best 3–4 fluorescent protein molecules per immobile focus, but likely to be substantially worse for Rep due to blurring of the fluorescent protein optical image in light of the rapid dynamics at the fork, which taken together explains the apparent appearance of lower stability foci reported in the earlier study. The cell strains we used in this study were reasonably healthy as assessed by cell doubling times in comparison to wild type and we could not detect any clear signs of filamentation defects whatsoever, which would if present of course be indicative of DNA damage and/or replication defects. We tagged both N- and C-termini of the proteins and evaluated them for functionality and chose alleles that do not have any obvious phenotypic differences from the wild type parent. The functionality of the fusions was tested by combining them pairwise with mutations that would render the strain inviable if the fusion was non-functional (these are shown in the plasmid loss assays of Supplementary Figure S2). The viability of the strains in these assays indicates that these fusions were definitely functional. However, when we tested for preservation of the functionality of mGFP-Rep in the presence of DnaQ-mCherry, we observed a reduction in the number of white plasmid-free colonies (this also is shown in Supplementary Figure S2) indicating a fitness cost of carrying multiple fluorescent tags. One explanation for this observation is that it may be attributed to increased transcription/translation rates as well as effects on protein folding kinetics (for example see (65)). The tagged protein may also display reduced mobility due to the bulky fluorophore adduct. Steric hindrance due to the attached fluorophore on the replisome components may result in suboptimal interaction of these proteins with other proteins at the heavily crowded multi-protein replisome. We also observed slightly delayed doubling times in these strains, compared to wild type. This was not unanticipated: genes encoding replisome components were similarly tagged with fluorescent proteins in earlier works too which exhibited signs of minimally increased growth rates (40,44). Thus, it is correct to surmise that these alleles are marginally compromised for their function, which may have ramifications on potential limitations for the interpretation of the data.

What are the implications of our data for the functioning of Rep as an accessory replicative helicase? Our data are consistent with Rep molecules bound to the DnaB hexamer associating continually with single-stranded DNA at the fork and translocating along this ssDNA in an ATP-dependent manner away from the replication fork. The 3′-5′ polarity of Rep translocation along ssDNA and the occlusion of the lagging strand template by the DnaB hexamer makes it likely that any Rep translocation will be along the leading strand template, consistent with Rep movement along this strand ahead of the fork to displace proteins out of the path of the advancing replisome (4,9). Such a model implies that at the majority of replisomes there is a continual firing of Rep molecules ahead of the replisome, analogous to bullets in a revolver. Having multiple Rep molecules translocating ahead of the fork might be needed for effective unwinding of double-stranded DNA and hence protein displacement ahead of the fork, given the inability of Rep monomers to unwind DNA *in vitro* in the absence of partner proteins (50,51). Indeed the stoichiometries we measure for Rep foci colocalized to the replisome lend support to the hypothesis that two Rep molecules may be acting in concert at the replication fork as well as six Rep molecules occupying the binding sites on the DnaB hexamer.

**Figure 4.**
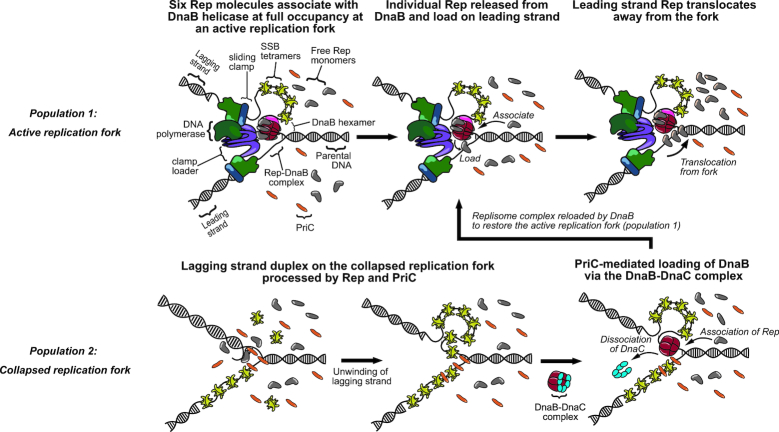
Model representing the two populations of Rep interactions Population1: Interaction of Rep with replicative helicase DnaB. Monomers of Rep (grey) associate with individual monomer subunits within the DnaB (red) hexamer to full occupancy of the hexamer. Rep monomers are continuously released from DnaB and load onto the leading strand. Released Rep monomers then translocate from the fork coupled to the hydrolysis of ATP. Additional Rep monomers from the cytoplasm are continuously recruited onto the DnaB hexamer as vacant binding sites become available. Rep can associate with PriC to stimulate replisome reloading. Population 2: A collapsed replication fork is recognized by PriC. DnaB is then loaded via the DnaB-DnaC complex. *Legend: DNA polymerase complex - green; sliding clamp – blue; clamp loader complex – purple; DnaB – red; DnaG – pink; single-strand binding protein (SSB) – yellow; Rep –grey; PriC–orange; DnaC–cyan*.

How does this model of accessory helicase activity interface with Rep acting as an accessory factor in PriC-catalyzed reloading of DnaB onto the lagging strand template during replication restart? The significant periodicity of numbers of Rep associated with the replisome depends on the Rep C-terminus rather than PriC (compare Figure 1F with Figure 2C and E), consistent with Rep association with the replisome being dominated by the Rep–DnaB interaction. However, the presence of PriC at 40% of forks leads to additional Rep molecules being associated with the replisome (Figure 2B). Colocalization of Rep with the replisome depends therefore upon both the Rep C-terminus and on PriC (Figure 2B). There are therefore two pools of Rep at the replisome, one pool dependent upon the Rep–DnaB interaction and another pool dependent on PriC (Figure 4). PriC interacts with single-stranded DNA and with SSB (66,67) providing means by which PriC could interact with the replisome and hence recruit Rep. Evidence for a direct Rep–PriC interaction is currently lacking but it is also possible that PriC recruits Rep to the replisome indirectly. Both PriC and Rep also interact with DnaB (9,25,68), and so association of PriC with DnaB might result in allosteric effects on DnaB that affect the known Rep–DnaB interaction (31). However, PriC is responsible for some colocalization of Rep with the replisome even in the Rep C-terminal mutant (Figure 2B) indicating that PriC-dependent recruitment of Rep is likely to be independent of any Rep–DnaB interaction.

Regardless of whether our observed PriC-dependent association of Rep with the replisome is a direct or indirect effect, our data lend support to a functional Rep–PriC interaction inside cells (29,30). Different dispositions of DnaB and PriC with respect to DNA within the replication fork might facilitate two different functions for the two different pools of Rep at the replisome. The DnaB-dependent pool of Rep very likely promotes replisome progression along protein-bound DNA via translocation of Rep along the leading strand template ahead of the fork (9). The second pool, associated directly or indirectly with PriC, might aid PriC-directed reloading of DnaB back onto the fork via Rep-catalyzed unwinding of the lagging strand duplex at the fork to generate single-stranded DNA for DnaB binding (30). Recruitment of Rep by two different factors at the replisome might therefore provide two ways in which Rep facilitates duplication of protein-bound DNA. However, the interplay between Rep and PriC is difficult to resolve. While PriC provides a pathway of replication restart, the accessory helicase function of Rep reduces the need for replication restart, complicating interpretation of this interplay. Our data do indicate, though, the importance of Rep and PriC for maintaining the architecture of the replisome. In both *repC4ala priC^+^* and *rep^+^ ΔpriC* cells the number of DnaQ molecules per focus is on average three, as found in wild type cells (40) (compare Figure 1C with Figure 2F and H). However, *repC4ala ΔpriC* cells have only 1–2 DnaQ molecules per focus (Figure 2G). This reduction in DnaQ molecules at the replisome is unlikely to be due to allosteric effects upon the structure of replisomes lacking Rep and PriC since replisomes that lack both Rep and PriC *in vitro* retain three DnaQ molecules (45). Alternatively this altered replisome architecture might be due to increased pausing and blockage of the replisome at nucleoprotein barriers in the absence of an accessory replicative helicase coupled with defective replisome reloading without PriC. Regardless of the reasons for this altered replisome structure, our data indicate that both Rep and PriC are important constituents of the replisome.

## DATA AVAILABILITY

Data included in full in main text and supplementary files. Raw data available from authors. Code written in MATLAB available at https://sourceforge.net/projects/york-biophysics/.

## Supplementary Material

gkz298_Supplemental_Files

## Notes

Present address: Jan-Gert Brüning, Molecular Biology Program, Memorial Sloan-Kettering Cancer Center, New York, NY 10065, USA.
