# Supplementary material for: Single-molecule live cell imaging of Rep reveals the dynamic interplay between an accessory replicative helicase and the replisome
Source: Nucleic Acids Res. 2019 Apr 27;47(12):6287–98. doi: 10.1093/nar/gkz298 (PMC6614839; doi:10.1093/nar/gkz298)
Supplement: gkz298_Supplemental_Files [file gkz298_supplemental_files.zip › Supplementary AppendixNAR_ML_mar2019_v25_no highlights.pdf]

Supplementary Information

Single-molecule live cell imaging of Rep reveals the dynamic interplay between an accessory replicative helicase and the replisome

Aisha H. Syeda[1,2], Adam J. M. Wollman[1,2], Alex L. Hargreaves[1,2], Jamieson A. L. Howard[1,2], Jan-Gert Brüning[2,3], Peter McGlynn[2,*] , Mark C. Leake [1,2,*]

The authors wish it to be known that, in their opinion, the first two authors should be regarded as joint First Authors

[1] Department of Physics, University of York, York YO10 5DD, United Kingdom.

[2] Department of Biology, University of York, York YO10 5DD, United Kingdom.

[3] Current address: Molecular Biology Program, Memorial Sloan-Kettering Cancer Center, New York, NY 10065, USA

* To whom correspondence should be addressed.

Tel: +44 (0)1904322697.Email: mark.leake@york.ac.uk or peter.mcglynn@york.ac.uk.

Present Address: Department of Physics and Biology, University of York, York YO10 5DD, United Kingdom

SI Table S1. Doubling times of labelled strains. Three replicates performed for each strain, OD measurements binned into 10 min interval time points.

**Strain (Relevant genotype)**	**Minimal medium**			**LB medium**		
	**Mean doubling time (min)**	**SD (min)**	**SEM (min)**	**Mean doubling time (min)**	**SD (min)**	**SEM (min)**
WT	80	10	5.8	23.3	5.8	3.3
repC4ala	93.3	5.8	3.3	40	0	0
rep2001	76.7	11.5	6.7	53.3	5.8	3.3
ΔpriC	106.7	11.5	6.7	46.7	5.8	3.3
dnaQ-mGFP	90	10	5.8	23.3	5.8	3.3
dnaQ-mCherry	93.3	5.8	3.3	26.7	5.8	3.3
mCherry-rep	96.7	5.8	3.3	30	0	0
mGFP-rep	93.3	5.8	3.3	36.7	5.8	3.3
mGFP-priC	103.3	5.8	3.3	53.3	5.8	3.3
ypet-dnaB	114.3	7.5	4.3	46.7	5.8	3.3
mGFP-repC4ala	113.3	5.8	3.3	36.7	5.8	3.3
mGFP-rep2001	113.3	11.5	6.7	60	10	5.8
dnaQ-mCherry mGFP-rep	96.7	11.5	6.7	23.3	5.8	3.3

dnaQ-mGFP mCherry-rep	96.7	11.5	6.7	26.7	5.8	3.3
dnaQ-mCherry mGFP-priC	123.3	5.8	3.3	40	10	5.8
dnaQ-mCherry mGFP-repC4ala	106.7	11.5	6.7	63.3	5.8	3.3
dnaQ-mCherry mGFP-rep2001	83.3	5.8	3.3	56.7	5.8	3.3
mCherry-rep ypet-dnaB	166.7	32.1	18.6	36.7	5.8	3.3
dnaQ-mCherry mGFP-repC4ala ΔpriC	103.3	15.3	8.8	53.3	5.8	3.3

SI Table S2: Strains used in this study

Strain	Relevant genotype	Source or derivation
BW25113 derivatives:		
BW25113	*rrnB ΔlacZ4787 hsdR514 Δ(araBAD)567 Δ(rhaBAD)568 rph-1*	(1)
JW0456	*ΔpriC752::<kan>*	(1)
JW5604	*Δrep729::<Kan>*	(1)
MG1655 derivatives:		
SS1076	*rep2001*	(2)
TB12	*ΔlacIZYA::<kan>*	
TB28	*ΔlacIZYA*	(3,4)
HB284	*ΔlacIZYA::<kan> rep2001*	SS1076 X P1(TB12)
TB28 derivatives		
N6556	*ΔlacIZYA::<> uvrD::dhfr Δrep::cat* / pAM403 (*rep*$^{+}$ *lacZ*$^{+}$, a pRC7 derivative)	(5)
N6568	*ΔlacIZYA::<> uvrD::dhfr* / pAM403 (*rep*$^{+}$ *lacZ*$^{+}$, a pRC7 derivative)	(5)
N6577	*Δrep::cat*	(5)
N7150	*ΔlacIZYA::<> rpoB*35 uvrD::dhfr Δrep::cat* / pAM407 (*uvrD*$^{+}$ *lacZ*$^{+}$, a pRC7 derivative)	(5)
HB139	*ΔpriC::<kan>*	TB28 x P1(JW0456) to Km^R

MKG3	*ΔpriC::<>*	HB139 to Km^{S} with pCP20
MKG10	*repΔC33-<kan>*	(6)
AM2017	*ΔlacIZYA ΔpriB::dhfr*	(7,8)
JGB129	*ΔpriC /* pKD46	MKG3 transformed with pKD46
JGB161	*ΔpriB::dhfr /* pAM421 (*priC*$^{+}$ *lacZ*$^{+}$, a pRC7 derivative)	AM2017 transformed with pAM421
JGB167	*ΔpriB ΔpriC /* pAM421 (*priC*$^{+}$ *lacZ*$^{+}$, a pRC7 derivative)	JGB161 x P1(JW0456) to Km^{R}
JGB255	*Δrep::apra*	*apra* integrated into AS97
JGB257	*Δrep::apra /* pKD46	JGB255 transformed with pKD46
JGB264	*mCherry-rep <Kan>*	*mCherry-rep <Kan>* recombineered into JGB257
JGB265	*mCherry-rep-<kan>*	TB28 x P1(JGB264) to Km^{R}
JGB266	*mCherry-Rep <Kan>*	TB28 x P1(JGB264) to Km^{R}
JGB286	*mCherry-Rep <>*	JGB266 to KmS with pCP20
JGB294	*dnaQ-mGFP-<kan> mCherry-rep-<>*	JGB286 x P1(AS217) to Km^{R}
JGB323	*ΔlacIZYA::<> repΔC33-<kan> ΔuvrD /* pAM403 (*rep*$^{+}$ *lacZ*$^{+}$, a pRC7 derivative)	N6568 x P1(MKG10)
KM269	*MG1655 ΔlacIZYA::<> rpoB*35 uvrD::dhfr Δrep729::<Kan>* / pAM407 (*uvrD*$^{+}$ *lacZ*$^{+}$, a pRC7 derivative)	N7150 x P1(JW5604) to Km^{R}

PM637	*rep2001*	HB284 to Km^{S} with pCP20
AS97	TB28/pKD46	TB28 transformed with pKD46
AS217	*dnaQ-mGFP-<kan>*	*mGFP-<kan>* with homology to dnaQ recombineered into AS97
AS446	*dnaQ-mCherry-<kan>*	*mCherry-<kan>* with homology to dnaQ recombineered into AS97
AS448	*dnaQ-mCherry-<>*	AS446 to km^{S} with pCP20
AS461	*ΔlacIZYA mGFP-priC-<kan>*	*mGFP-priC-<kan>* recombineered into JGB129
AS476	JGB161 *mGFP-priC-<kan>*	JGB161 x P1(AS461) to Km^{R}
AS488	*dnaQ-mCherry-<> mGFP-priC-<kan>*	AS448 x P1(AS461) to Km^{R}
AS505	JGB257 *mGFP-rep-<kan>*	*mGFP-rep-<kan>* recombineered into JGB257
AS510	*ΔlacIZYA::<> mGFP-rep-<kan> ΔuvrD /* pAM403 (*rep*$^{+}$ *lacZ*$^{+}$, a pRC7 derivative)	N6568 x P1(AS505)
AS525	*mGFP-rep-<kan>*	TB28 X P1(AS505) to Km^{R}
AS545	*dnaQ-mCherry-<> mGFP-rep-<kan>*	AS448 X P1(AS505) to km^{R}
AS608	*mGFP-repΔC33-<kan>*	*mGFP-repΔC33* *<Kan>* recombineered into JGB257
AS609	*mGFP-repC4ala-<kan>*	*mGFP-repC4ala-<kan>* recombineered into JGB257
AS615	*dnaQ-mCherry-<> mGFP-repC4ala-<kan>*	AS448 X P1(AS609) to Km^{R}

AS622	*ΔlacIZYA::<> mGFP-repC4ala-<kan> ΔuvrD* / pAM403 (*rep*$^+$ *lacZ*$^+$, a pRC7 derivative)	N6568 x P1(AS609)
AS625	*ΔlacIZYA::<> mGFP-repΔC33-<kan> ΔuvrD /* pAM403 (*rep*$^+$ *lacZ*$^+$, a pRC7 derivative)	N6568 x P1(AS608)
AS662	*dnaQ-mCherry-<> mGFP-rep-<> ΔpriC::<kan>*	AS630 X P1(JW0456) to Km^R
AS666	*dnaQ-mCherry-<> mGFP-repC4ala-<>*	AS615 to km^S with pCP20
AS672	*dnaQ-mCherry-<> mGFP-repC4ala-<> ΔpriC::<kan>*	AS666 X P1(JW0456) to Km^R
AS674	*mGFP-rep2001-<kan>*	*mGFP-rep2001-<kan>* recombineered into JGB257
AS690	*dnaQ-mCherry-<> mGFP-rep2001-<kan>*	AS448 X P1(AS674)
AS857	*repC4ala-<kan>*	*repC4ala-<kan>* recombineered into AS97
AS870	*dnaQ-mCherry-<> mGFP-rep-<kan> /* pAM407	AS545 transformed with pAM407
AS874	*dnaQ-mCherry-<> mGFP-rep-<kan> ΔuvrD::dhfr /* pAM407	AS870 X P1(N6568)
AB1157 derivatives		
RRL368	*<>-ypet-dnaB*	(9)
AS860	*mCherry-rep-<kan> <>-ypet-dnaB*	RRL368 X P1(JGB264)
BL21 derivatives		
HB222	*Δrep::cat*	BL21 AI x P1(N6577) to Cm^r

SI Table S3. Plasmids used in this study

**Plasmid**	**Description**	**Antibiotic**	**Reference**
pCP20	Yeast Flp recominase expression plasmid	Amp	(10)
pDHL580	pUC19 *linker-mGFPmut30<kan>*	Amp, kan	(11)
pKD46	λ Red recombinase expression plasmid	Amp	(10)
pAS65	pUC18 *mGFP-priC-<kan>*	Amp, kan	This study
pAS79	pUC18 *mGFP-rep-<kan>*	Amp, kan	This study
pAS124	pUC18 *mGFP-repC4ala-<kan>*	Amp, kan	This study
pAS127	pUC18 *mGFP-rep2001-<kan>*	Amp, kan	This study
pJGB374	pUC18 *linker-mCherry-<kan>*	Amp, kan	This study
pJGB380	pUC18 *mCherry-rep-<kan>*	Amp, kan	This study
pPM638	pBAD24 *kan*	Kan	(5)
pPM648	pBAD*rep*	Kan	(5)
pPM816	pBAD*repG672A,K673A*	Kan	This study
pPM817	pBAD*repK670A,R671A*	Kan	This study
pMG41	pBAD*repC4Ala*	Kan	This study
pJLH237	pET14b *mGFP-rep*	Amp	This study
pJLH238	pET14b *mGFP-repC4ala*	Amp	This study

SI Table S4. Primers used in this study

Primer	Sequence (5' - 3')
oAS77	CCCGTCTCGATCTGGTGCAGAAGAAAGGCGGAAGTTGCCTCTGGCGAGCAGCC AGTGAATTCGAGCTCAG
oAS79	ACGATCTCCGTGGCCTCATTGGTTCGGAGCAGGTGGAACTGGAGTTTGACGCCA GTGAATTCGAGCTCAG
oAS84	CCCGTCTCGATCTGGTGCAG
oAS85	TTGCTGCAAAAATCGCCCAAG
oAS132	CCCGTCTCGATCTGGTGCAGAAGAAAGGCGGAAGTTGCCTCTGGCGAGCAGGC TGGCTCCGCTGCTGG
oAS133	TTGCTGCAAAAATCGCCCAAGTCGCTATTTTTAGCGCCTTTCACAGGTATCATAT GAATATCCTCCTTAG
oAS136	AGTGGCTACTTTAGCATAACAATTATCATTTTCAATGAGGTCTTATCATGAGTAAA GGAGAAGAACTT
oAS141	GATTCTGCTACAATCCTCCCCCCGTTCGAAGATTGAGCAATACACCTATGAGTAA AGGAGAAGAACTT
oJGB379	GATTCTGCTACAATCCTCCCCCCGTTCGAAGATTGAGCAATACACCTATGGTGAG CAAGG
oJGB380	TTAATGAGTAAGTGCCGGATGCGATGCTGACGCATCTTTTCCGGCCTTGACATAT GAATATCCTCCTTAG
oJGB389	CCAGTTTGACATCGTCAGGGGCATTTTCCAGTGACATATTCTCTCCATTGCATAT GAATATCCTCCTTAG

oJGB402	GGAAGAACGGGCAAAGCACC
oJGB403	CCTTAGGGGACATTTAGCGAC
oJGB417	TACAAGACACGTGCTGAAGTC
oJGB418	TGCTAGTTGAACGCTTCCATC
oMKG70	CAGTCATAGCCGAATAGCCT
oMKG71	CGGTGCCCTGAATGAACTGC
oPM187B20	GTCGGATCCTCTAGACAGC(biodT)CCATGATCACTGGCACTGGTAGAATTCGGC
oPM188B34	AACGTCATAGACGATTACATTGCTACATGGAGC(biodT)GTCTAGAGGATCCGAC
oPM363	CATACGTTGGGGCTGGAT
oPM369	TCGATGAAGATAATATCG
oPM372	GTTGGTTGACGTTCTTCA
oPM376	GTGTGCATCATACAGCCC

SI Table S5. Number of cells and trajectories analysed

Strain	Number of trajectories	Number of colocalized trajectories	Number of cells
mGFP-rep	924	367	38
mGFP-rep ΔpriC	1054	354	41
mGFP-rep2001	884	271	36
mGFP-repC4Ala	655	267	50
mGFP-repC4Ala ΔpriC	89	42	54

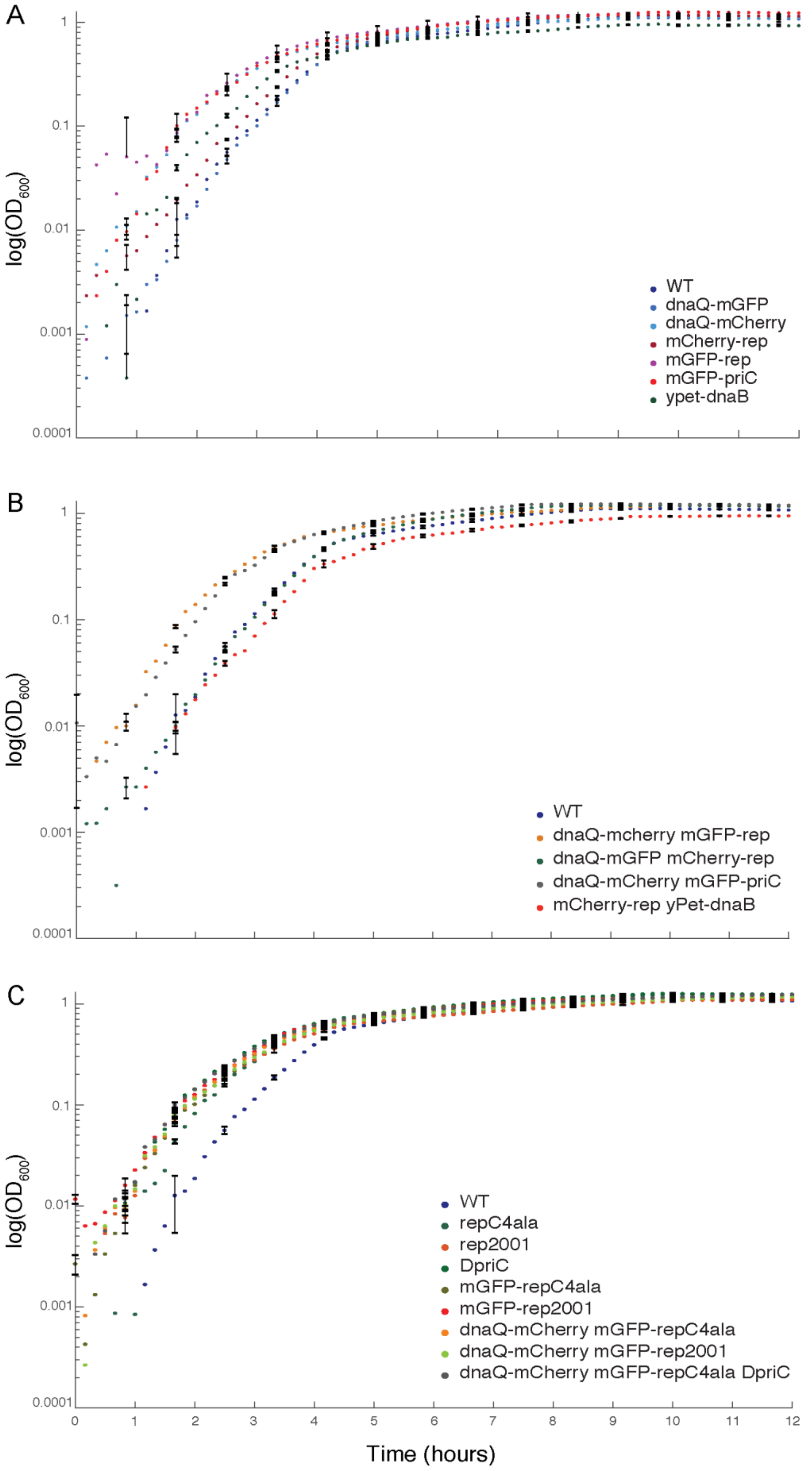
A
B
C
log(OD600)
1
0.1
0.01
0.001
0.0001
0 1 2 3 4 5 6 7 8 9 10 11 12
Time (hours)
WT
dnaQ-mGFP
dnaQ-mCherry
mCherry-rep
mGFP-rep
mGFP-priC
ypet-dnaB
dnaQ-mcherry mGFP-rep
dnaQ-mGFP mCherry-rep
dnaQ-mCherry mGFP-priC
mCherry-rep yPet-dnaB
repC4ala
rep2001
DpriC
mGFP-repC4ala
mGFP-rep2001
dnaQ-mCherry mGFP-repC4ala
dnaQ-mCherry mGFP-rep2001
dnaQ-mCherry mGFP-repC4ala DpriC

Figure S1. Growth curves in LB medium. Cultures were grown in LB as described in the text. The OD_{600} values are plotted on a log scale on the vertical axis with a linear scale of time in hours on the horizontal axis.. The relevant genotypes of the strains are indicated above the respective curves. SD errorbars (shown just on every 5th consecutive point for clarity here), taken from N=3 replicate cultures. Plots in panel A are growth curves of single labelled wild- ype (WT) strains, those in panel B are dual labelled wild-type strains, while panel C shows growth curves of all mutant strains. The unlabelled wild type strain is included in all panels as a reference.

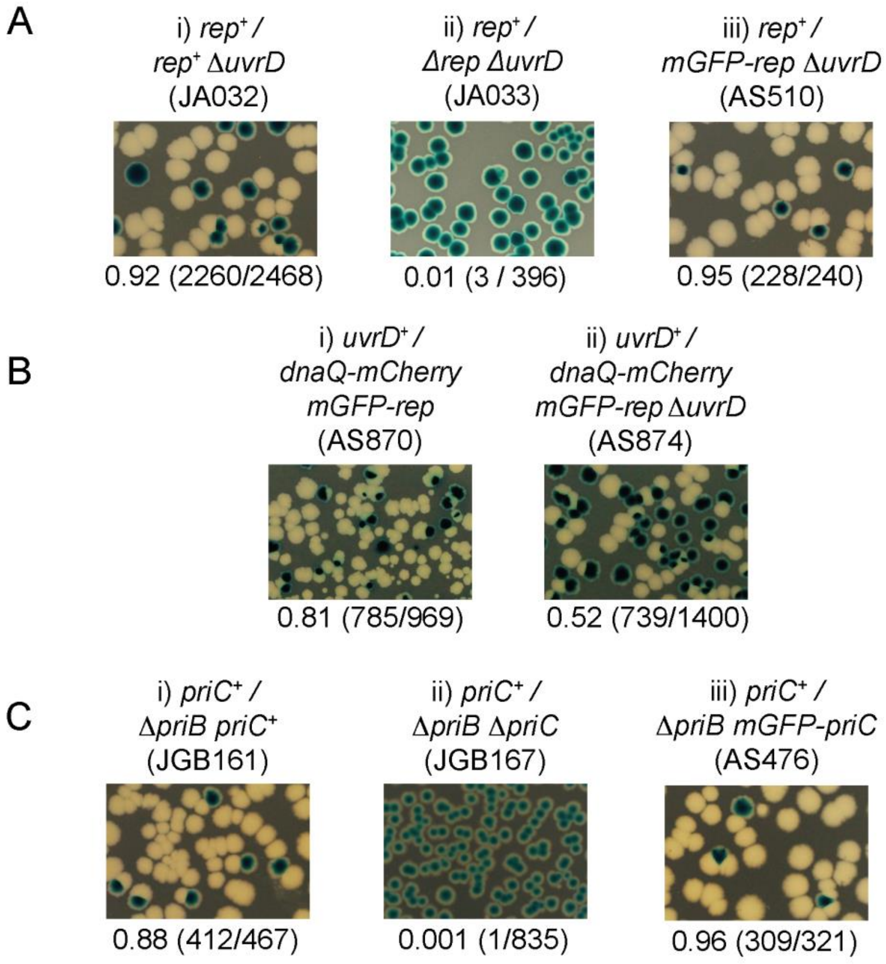

Figure S2. Testing of mGFP-Rep and mGFP-PriC fusions for retention of function. A. We transformed the strain carrying a chromosomal *mGFP-rep* allele with a derivative of pRC7 carrying a wild-type *rep* allele (pAM403), which is an unstable low copy plasmid that also carries the *lacZYA* genes (4). Presence of this plasmid confers blue colour to the colonies on Xgal indicator plates in strains chromosomally deleted for the *lac* operon. In rapidly growing cells, *rep* or *uvrD* is essential for viability, while loss of both is inviable (5,12). Therefore, *ΔuvrD* cells with a functional *rep* allele are viable and can readily lose pRC7*rep*, giving rise to white colonies on LB Xgal IPTG plates, but cells lacking *rep* function cannot lose pRC7*rep* (5,13) (see Ai and ii). The *mGFP-rep ΔuvrD* cells produced white colonies on Xgal media, indicating that the *mGFP-rep* fusion was functional (see Aiii). B. We tested for functionality of the *mGFP-rep* fusion allele in the strain carrying *dnaQ-mCherry.* This strain

carried a derivative of pRC7 carrying a wild type *uvrD* allele (pAM407). Loss of the plasmid and recovery of healthy plasmid-free white colonies in a *ΔuvrD* background indicated that *mGFP-rep* was functional in this strain. C. We also generated an *mGFP-priC* fusion and tested for retention of function by introducing a pRC7 derivative carrying the wild-type *priC* allele (pAM421) in a *ΔpriB ΔlacZYA* strain. Cells require either functional PriB or PriC for viability but loss of both is lethal (14) (See Bi and ii). *mGFP-priC* Δ*priB* cells could lose pRC7*priC* indicating a functional PriC fusion protein (See Biii). The fractions under the pictures denote the proportion of white colonies to the total number of colonies obtained. The numerator in the parentheses indicates the number of white colonies observed while the denominator indicates the total number of colonies observed.

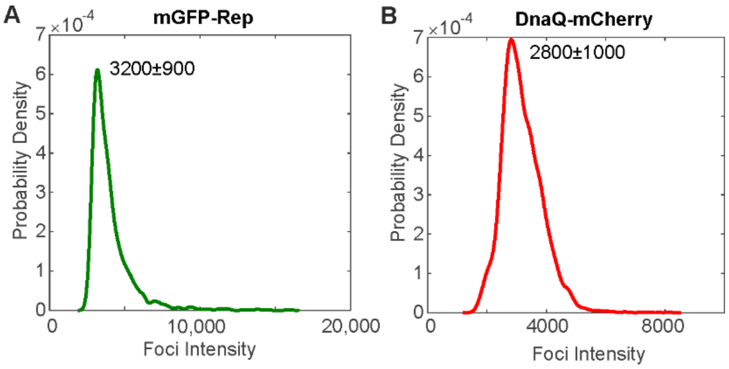

Figure S3. Brightness of single mGFP and mCherry molecules A. and B. Characteristic intensity distributions rendered as kernel density estimates of single mGFP-Rep and DnaQ-mCherry. Peak±full width at half maximum indicated. Distributions calculated from the tracked foci intensity distributions from the end of the photobleach process such that only single fluorophore molecules are detected. Number of molecules per focus before bleaching is determined by dividing the initial focus intensity by these values for the equivalent fluorophore.

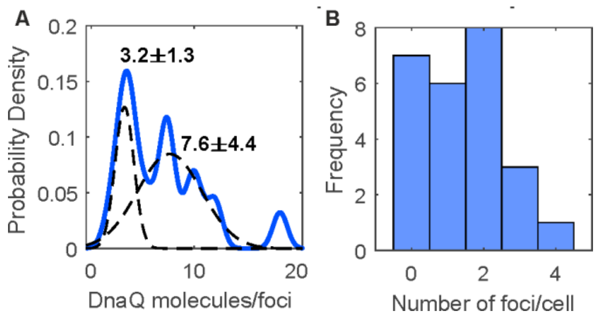

Figure S4. DnaQ-mGFP Slimfield analysis. A. KDE of DnaQ foci stoichiometry with double Gaussian fits in dotted lines, peak values ± SE indicated. B. Histogram showing the number of DnaQ foci detected per cell.

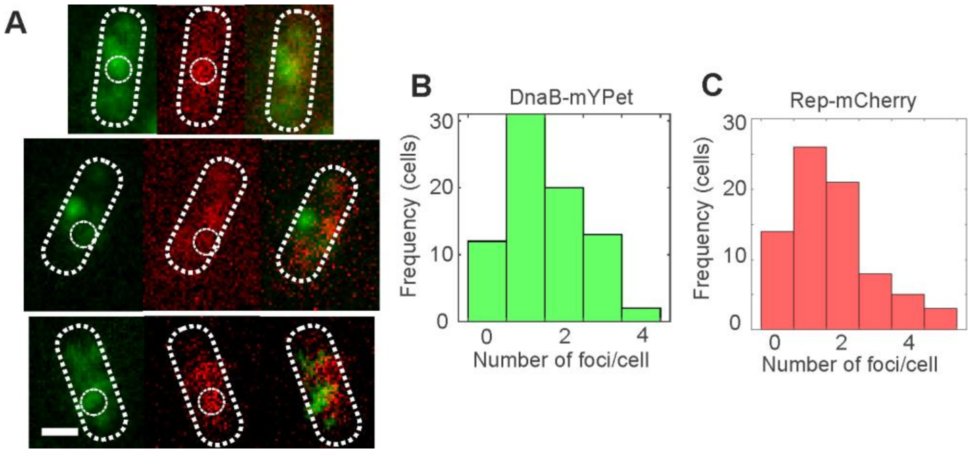

Figure S5. Dual colour imaging Rep and DnaB. A. Dual colour Slimfield images of DnaB-mYPet:Rep-mCherry with example detected colocalized foci marked with white circles. Scale bar 1 micron. B. Number of detected DnaB foci/cell C. Number of detected Rep foci/cell (these data use a threshold of 2 consecutive image frames for foci track acceptance, compared to the default of 4, to correct for differences in the photobleaching rates of mYPet compared to mCherry); N=77 cells.

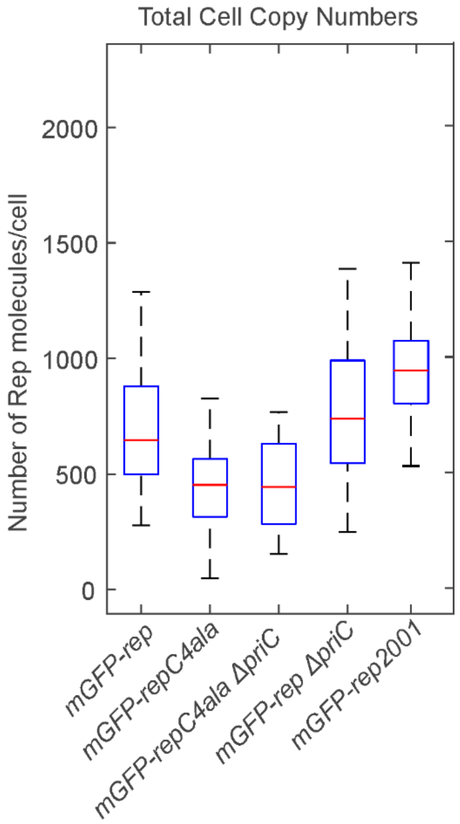

Figure S6. Total cell copy numbers. Boxplot of the total number of mGFP-Rep molecules per cell, estimated by numerical integration of the whole cell fluorescence. Median is shown in red, bottom and top of the blue box mark the 25th and 75th percentiles and whiskers extend to the most extreme points not considered outliers (2.7 standard deviations covering 99.3% of normally distributed data).

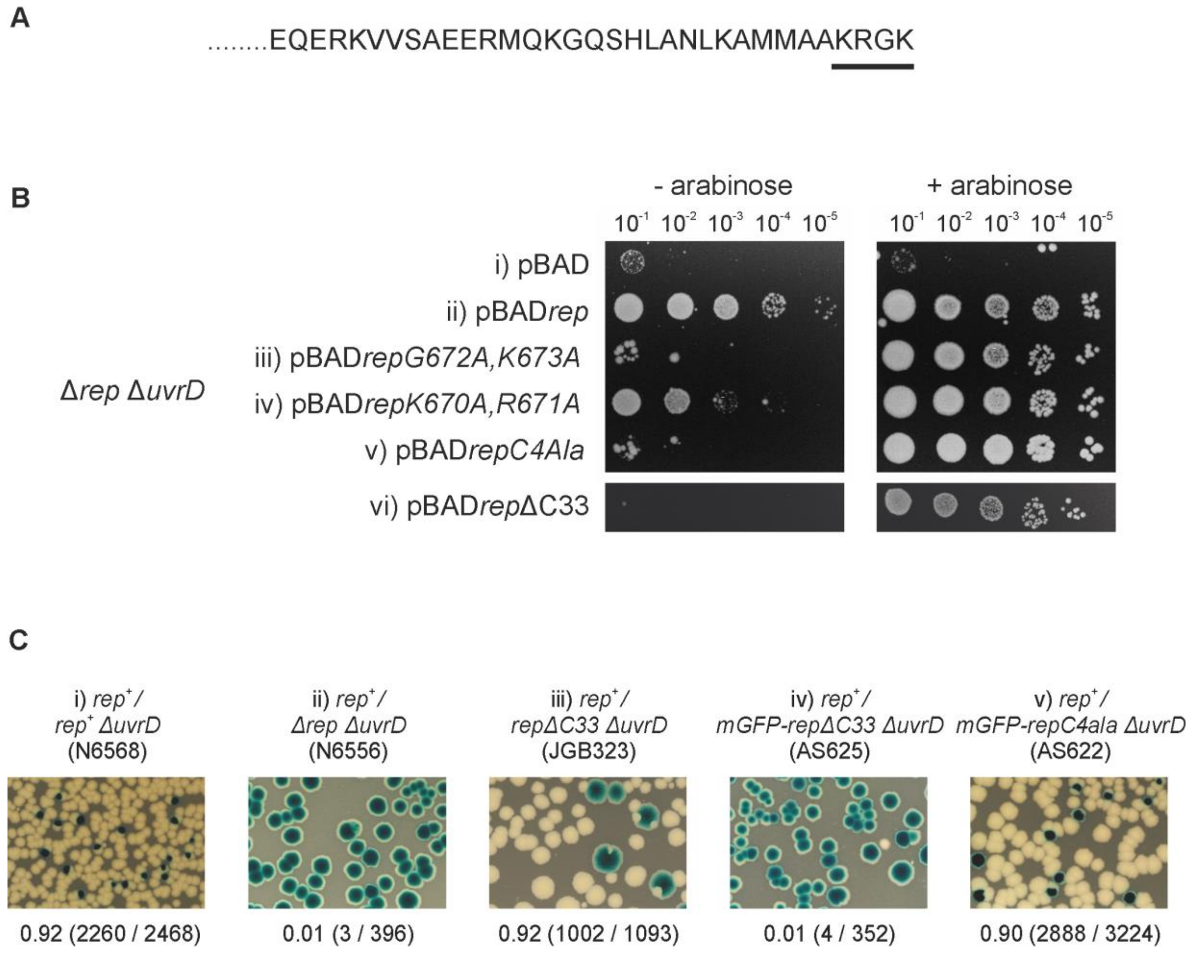

Figure S7. Generation of a *rep* mutant that phenocopies *repΔC33* but which does not lose function when fused to mGFP. A. The C-terminal 33 amino acids of Rep within which the DnaB interaction domain resides (5). The final four residues of this C-terminal region, underlined, were chosen as targets for mutagenesis based on their unusually high charge density. B. The plasmid pBAD bears an arabinose-inducible promoter that provides very low and very high levels of expression of genes when arabinose is absent or present in the growth medium, respectively (5). Strains lacking both the *rep* and *uvrD* genes are inviable when grown rapidly on rich medium (5,12) and so Δ*rep* Δ*uvrD*/pBAD cells cannot form colonies on LB agar due to absence of a complementing helicase gene in pBAD (5) (see also i). In contrast, pBAD*rep*, encoding wild type *rep*, allows Δ*rep* Δ*uvrD* cells to grow on LB agar in both the absence and presence of arabinose, consonant with very low levels of *rep* gene expression being sufficient to sustain viability (5) (see also ii). Absence of the Rep-DnaB interaction is characterised by loss of complementation at very low levels of helicase expression (- arabinose) but maintenance of complementation at high expression levels (+ arabinose) (5). We used this pattern of complementation as a readout of the interaction between Rep and DnaB.

We introduced pairs of alanine mutations into the final four codons within pBADrep. Both pBAD*repG672A, K673A* and pBAD*repK670A, R671A* displayed reduced complementation in the absence of arabinose but full complementation with arabinose, indicating involvement of both pairs of residues in the Rep-DnaB interaction (iii and iv). We therefore constructed pBAD*repC4Ala*, in which all four C-terminal residues are mutated to alanine and found that complementation required arabinose (Bv). These data indicate that the final 4 amino acids within the Rep C-terminal region are the residues that determine the phenotype displayed by *rep*Δ*C33* (compare v and vi). C. To determine whether an *mGFP-repC4Ala* fusion retains function, we employed a plasmid loss assay to determine the viability of strains. pRC7 is a highly unstable, very low copy plasmid that encodes *lacIZYA* (4). Retention or loss of this plasmid can be monitored by blue/white colony colour in strains bearing a chromosomal deletion of the lac operon. pAM403 is a derivative of pRC7 encoding wild type *rep* (15). *rep*$^{+}$ Δ*uvrD* cells can lose pRC7*rep* rapidly under rapid growth conditions, forming white colonies on LB X-gal IPTG plates, whereas Δ*rep* Δ*uvrD* cells can grow only if they retain pRC7*rep* (5,13) (see also Ci and ii). *repΔC33* Δ*uvrD* cells are viable since native expression levels of *repΔC33* are sufficient to retain partial accessory helicase function (2,16). However, fusion of *repΔC33* to *mGFP* resulted in much lower viability than the original *rep*Δ*C33* allele (compare iv with iii). In contrast, *mGFP-repC4Ala* Δ*uvrD* cells retained viability indicating that the mGFP fusion did not have an adverse effect on RepC4Ala function (v). The fractions under the pictures denote the proportion of white colonies to the total number of colonies obtained. The numerator in the parentheses indicates the number of white colonies observed while the denominator indicates the total number of colonies observed.

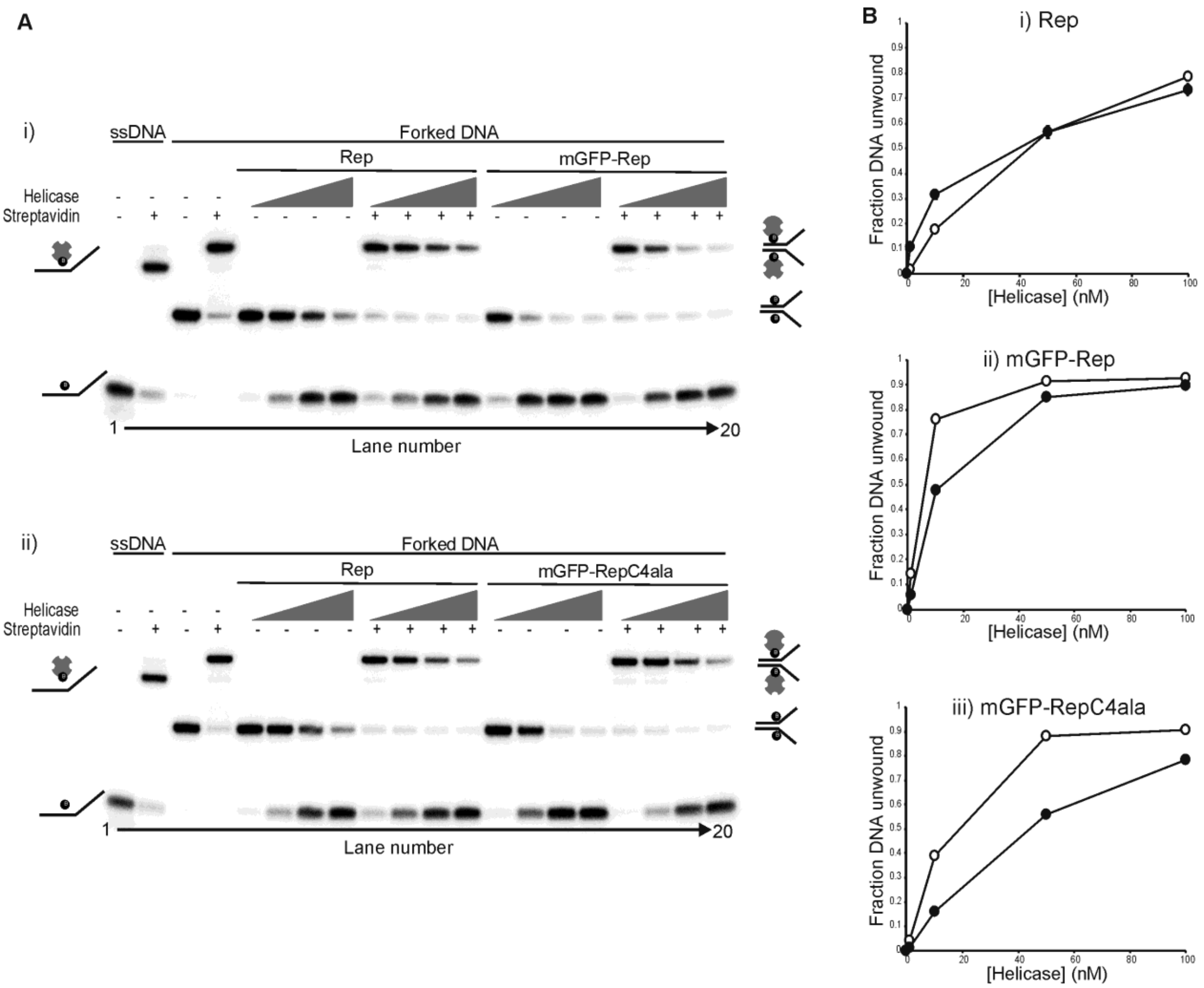

Figure S8. mGFP-Rep fusions are functional *In vitro*. A. Native polyacrylamide TBE gels showing Rep and mGFP-Rep (i) or Rep and mGFP-RepC4ala (ii) unwinding of forked DNA containing biotin on both strands within the duplex region (17,18). Lanes 1-4 contain markers indicating the position of single stranded or forked DNA +/- streptavidin as indicated. Lanes 5-20 contain the products of unwinding the forked DNA +/- streptavidin by the indicated helicase at 1, 10, 50 and 100 nM. **B.** Quantification of the unwinding of the forked substrate in the absence of (open circles) and presence of (closed circles) streptavidin by the indicated helicases.

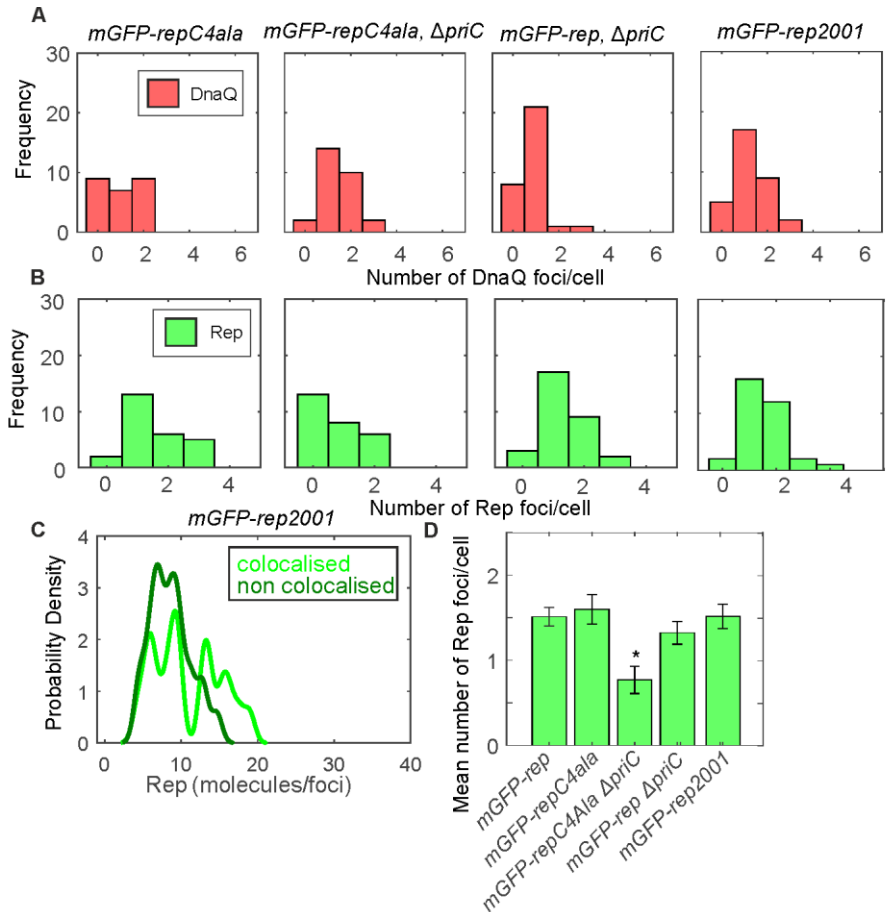

Figure S9. Colocalization analysis of Rep mutants. A. Number of detected DnaQ foci/cell and B. Number of detected Rep foci/cell in the absence and presence of *repC4Ala*, Δ*priC* and *rep2001* C. Kernel density estimates of the number of mGFP-Rep molecules in foci colocalized with DnaQ-mCherry (light green lines) and foci not colocalized with DnaQ-mCherry (dark green lines) in *mGFP-rep2001*. D. The mean number of mGFP-Rep foci detected per cell for wild type and mutant strains. SE indicated.

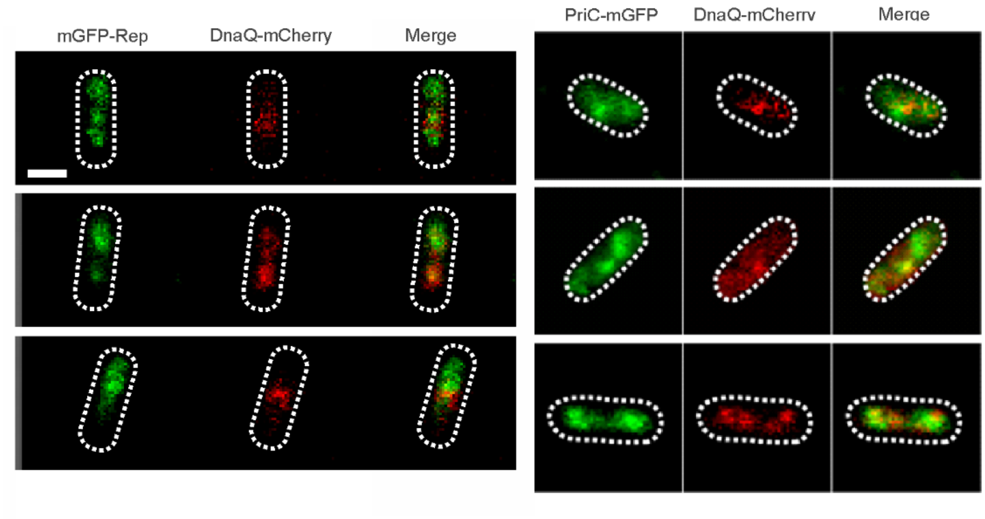

Figure S10. Rep and PriC localization. Dual colour Slimfield images of mGFP-Rep:DnaQ-mCherry (left panel) and mGFP-PriC:DnaQ-mCherry (right panel), scale bar 1 micron.

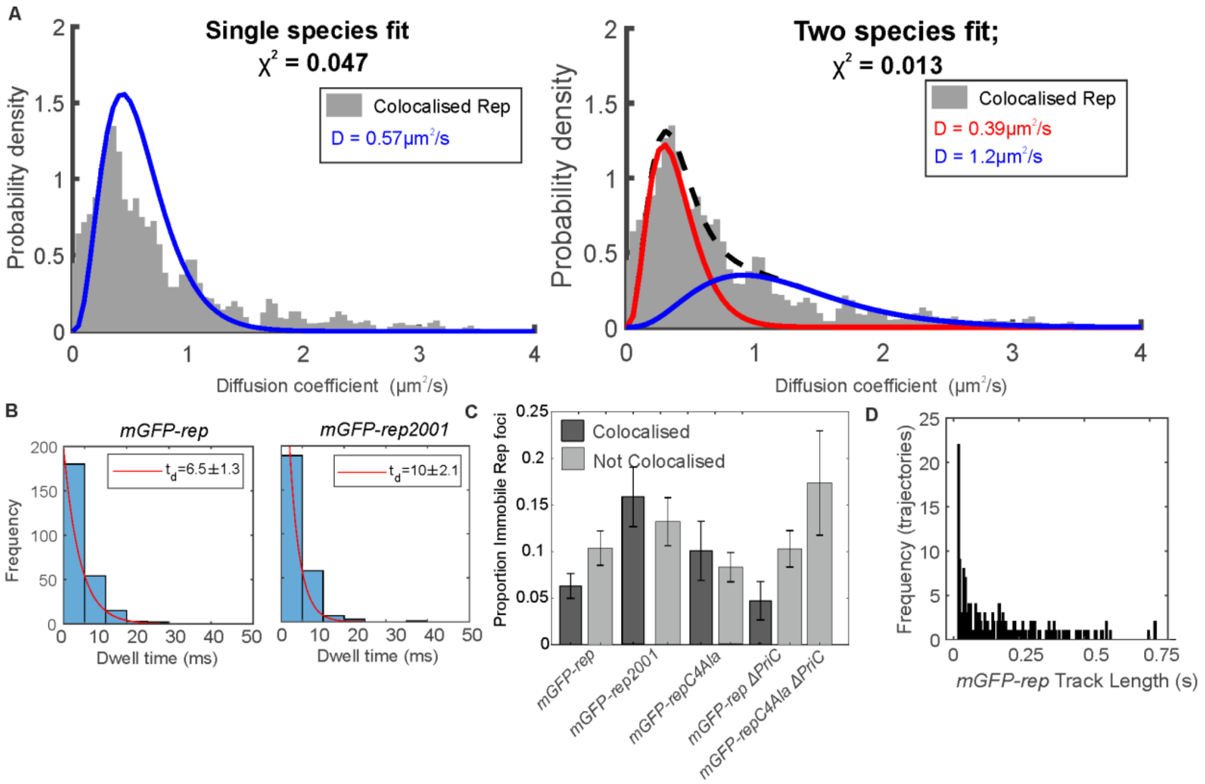

Figure S11. Mobility analysis of Rep mutant. A. 1 and 2 species (diffusion coefficients in this case) fits to colocalized Rep diffusion coefficients with larger reduced chi^2 than the 3 species fit shown in Fig. 3. Thus 3 species fits were used for all the data. B. mGFP-Rep foci dwell time with mCherry-DnaQ foci distribution with an exponential fit (red) in the absence and presence of *rep2001* C. Proportion of immobile colocalized and non-colocalized mGFP-Rep in the absence and presence of *repC4Ala*, Δ*priC* and *rep2001.* Error bars given by the 95% confidence intervals of the fit. D. Histogram of mGFP-rep track length.

SI Text

Strain construction

All strains used in this study are derivatives of the laboratory wild-type strain TB28. Briefly, for tagging *dnaQ*, *linker-mGFPmut3* followed by a kanamycin resistance cassette flanked by *frt* sites was amplified by PCR from the plasmid pDHL580 (11) using primers oAS77 and oAS79 (SI Table S4), and *linker-mCherry-<kan>* was amplified from pJGB374 using primers oAS132 and oAS133. The amplification primers had a 50 bp homology at their 5’ end to the last 50 bp of the *dnaQ* gene preceding the stop codon (forward primer) or the 50 bp immediately after the stop codon (reverse primer). The resulting PCR products thus had homology either side such that recombination with the chromosome would result in in-frame integration of *linker-mGFP-<kan>* and *linker-mCherry-<kan>* immediately downstream of *dnaQ*, resulting in *dnaQ- mGFP-<kan>* and *dnaQ- mCherry-<kan>* alleles.

The PCR products were treated with *Dpn*I, gel purified, and introduced by electroporation into cells expressing the lambda Red genes from the plasmid pKD46 (10). The recombinants were selected for kanamycin resistance and screened for ampicillin sensitivity. The colonies obtained were verified for integration by PCR and sequencing with primers oAS84 and oAS85.

mGFP-rep-<kan> fusions for various *rep* alleles were amplified from plasmids pAS79 (*rep*$^{+}$), pAS124 (*repC4ala*) and pAS127 (*rep2001*) with primers oAS141 and oJGB380 having 50 bp homology on either end of the native *rep* locus. Likewise *mCherry-rep-<kan>* was amplified from pJGB380 using primers oJGB379 and oJGB380. *mGFP-priC-<kan>* was amplified from the plasmid pAS65 using primers oAS136 and oJGB389. All PCR products were introduced on the chromosome of cells expressing lambda red genes at the native loci after *Dpn*I digestion, gel extraction, and electroporation as described above for *dnaQ* fusions. The *rep* recombinants were verified by PCR amplification and sequencing using the primers oJGB418, oMKG70, oMKG71, oPM363, oPM372, and oPM376. The *priC* recombinants were verified by PCR amplification and sequencing with primers oJGB402, oJGB403, oJGB417, and oJGB418.

Where required, the kanamycin resistance gene was removed by expressing Flp recombinase from the plasmid pCP20 (10) to generate kanamycin sensitive strains carrying the FP fusions.

Dual labelled strains were created by introducing the kanamycin tagged FP alleles by standard P1 mediated transduction into single labelled strains carrying the required FP allele after removing the linked kanamycin marker.

All plasmids used in this study are listed in Table S3 and all primers are listed in Table S4.

<u>RepC4Ala</u>

pBAD is a plasmid conferring kanamycin resistance that contains an arabinose-inducible promoter upstream of a multiple cloning site whilst pBAD*rep* is a derivative encoding wild type Rep (5). pBAD*repG672A,K673A* and pBAD*repK670A,R671A* were constructed by site-directed mutagenesis of the indicated codons within pBAD*rep*. pBAD*repC4Ala* is a derivative of pBAD*rep* in which all four codons were altered by site-directed mutagenesis to encode alanine. Assays to determine the ability of pBAD and derivatives to complement *Δrep ΔuvrD* inviability on rich medium were performed as described (5). Plasmid loss experiments to determine the viability of combinations of chromosomal alleles were performed as described (15).

<u>Determination of generation time of the *E. coli* strains by analysis of growth:</u>

Cells were grown overnight in LB medium at 37°C at 200rpm. The saturated overnight cultures were diluted 100 fold into fresh LB or washed once with 1X 56 salts and diluted 100 fold in fresh 1X 56 salts with 0.2% glucose as the carbon source. Aliquots of 100 µl each of the diluted cultures in the fresh media were pipetted into individual wells of 96 well clear flat bottom sterile microplates (Corning). The microplates containing the diluted cultures was incubated in a BMG LABTECH SPECTROstar Nano microplate reader at 37°C and the optical density (A600) values were recorded at defined time intervals. The time taken for the optical density values to double during the exponential growth phase of the culture was taken as the generation time. The values expressed are the means of three independent replicates, with the standard deviation and standard errors indicated.

Single-molecule microscopy and analysis

A dual colour bespoke single-molecule microscope was used (19) which used a narrow 10µm at full width half maximum excitation field at the sample plane to generate Slimfield illumination. Excitation was from 488nm and 561nm 50mW Obis lasers digitally modulated to produce alternating laser excitation with 5ms period. Modulation was produced by National Instruments dynamic I/O module NI 9402. Excitation was coupled into a Zeiss microscope body with a Mad City Lab's nanostage holding the sample. Emission was magnified to 80nm/pixel and imaged using an Andor Ixon 128 emCCD camera. Green/Red images were split using a bespoke colour splitter consisting of a dual-pass green/red dichroic mirror centred at long-pass wavelength 560nm and emission filters with 25nm bandwidths centred at 542nm and 594nm.

Samples were imaged on agarose pads suffused with media as described previously (20).

Foci were automatically detected and tracked using bespoke MATLAB software described previously (21). In brief bright foci were identified by image transformation and thresholding. The centroid of candidate foci were determined using iterative Gaussian masking (22) and accepted if their intensity was greater than a signal to noise ratio (SNR) of 0.4. Intensity was defined as the summed pixel intensity inside a 5 pixel circular region of interest (ROI) corrected for the background in an outer square ROI of 17x17 pixels. SNR was defined as the mean BG corrected pixel intensity in the circular ROI divided by the standard deviation in the square ROI. Foci were linked together into trajectories between frames if they were within 5 pixels of each other. Linked foci were accepted as "tracks" nominally if they persist for at least 4 consecutive image frames, unless specified otherwise.

Stoichiometry was determined by fitting the first 3 intensity values of a foci to a straight line, using the intercept as the initial intensity and dividing this by the characteristic intensity of GFP or mCherry. This characteristic intensity was determined from the distribution of foci intensity values towards the end of the photobleach confirmed by overtracking foci beyond their bleaching to generate individual photobleach steps of the characteristic intensity (Fig S2). The number of peaks in the Gaussian fits to Rep was set by running a peak fitting algorithm over the wild type distribution. This number of Gaussians was then used for mutant distributions unless two or more of the Gaussians

converged on the same/similar peak value, in which case they were removed. For DnaQ, two peaks were fit as used previously (23).

Red and green images were aligned based on the peak of the 2D cross correlation between brightfield images. Colocalization between foci and the probability of random colocalization was determined as described previously (24).

Microscopic diffusion coefficients were calculated by fitting the first 3 mean square displacement (MSD) values, i.e. equivalent to time interval values of 5, 10 and 15 ms, with a linear fit constrained through the equivalent localization precision MSD (25). Dwell time was calculated as the number of frames that each trajectory was colocalized with the fork position, as determined by the DnaQ foci detected at time zero.

The upper bound of stoichiometry in the pool was calculated using an approach modified from previously (19). We modelled an average *E. coli* cell volume as equivalent to a cylinder of diameter 1 µm and length which varies between 1-4 µm depending on the stage in the cell cycle, capped by 2 hemispheres (26). This morphology indicates a mean volume *V* of 3.7-13.1 μm^3 per cell, assumed largely accessible to Rep unlike far large protein complexes such as polysomes which exhibit nucleoid exclusion (27). If the mean cell copy number in the pool for Rep is *n* with mean foci stoichiometry of *S* then the mean number of Rep foci *F* in the pool is *n/S*. If each Rep focus occupies an equivalent sphere of radius r such that the sum of all spheres is equivalent to the cell volume then $F.4\pi r^3/3=V$. The optical resolution limit, identified as the pointed spread function width *w* of our microscope, for our setup was measured previously for mGFP excitation to be ~230 nm (19). For Rep foci to be part of the pool implies that the mean nearest neighbour foci separation (i.e. 2*r*) is not greater than *w*, such that *r* is the radius of the sphere associated with each focus with the sum of all such spheres having a total volume *V*. Thus, assuming relative insensitivity to blur artefacts(4) with our rapid sampling:

$$2r \leq w$$

$$V = F\left(\frac{4}{3}\pi r^3\right)$$

$$\therefore r = \sqrt[3]{\left(\frac{3SV}{4n\pi}\right)} \leq w/2$$

$$\therefore S \leq \frac{nw^3\pi}{6V}$$

Using an average value of *n* of ~650 molecules per cell (SI Fig. 7) the range in *V* suggests an upper limit to *S* in the range 0.3-1.1 molecules per Rep focus, consistent with a monomeric pool for Rep, to the nearest integer.

The mean nearest neighbour separation for Rep monomers in this cytoplasmic pool, assuming a typical cell length of ~3µm is ~100 nm. This means that within the diffraction-limited image of a detected Rep hexamer, which is defined by the optical resolution limit of ~230 nm, roughly two monomeric mGFP-Rep molecules on average will also be present, and so the apparent absolute value of stoichiometry of the Rep hexamer (or of integer numbers of overlapping Rep hexamer images) may be greater than that expected from hexameric periodicity by up to ~2 molecules depending upon how uniform or not the real pool background actually is which is borne out also when we run image simulations through the same foci tracking and analysis software using realistic levels of signal and noise for foreground hexameric Rep complexes and background diffusive monomeric Rep, This is very close to what we observe for the stoichiometry distribution of mGFP-Rep (see Fig. 1F).

Dual labelled Rep/DnaB

Preliminary attempts to construct a DnaB-mCherry fusion resulted in non-viable filamentous cells. However, we managed to construct a viable non-filamentous strain (Table S1) using an existing strain which contained a mYPet-DnaB fusion (9) into which we then moved the mCherry-Rep fusion. This resulted in resolvable DnaB-mYPet and Rep-mCherry foci, albeit with less optimal photophysical properties compared to mGFP/mCherry imaging due to the higher peak emission wavelength of mYPet compared to mGFP and relative dimness and photo-instability of mCherry compared to mYPet, but still indicating similar numbers of foci per cell as measured for the DnaQ replication fork marker in our other Rep/DnaQ strains (Fig S5). Applying criteria such that foci were accepted with only 2

consecutive image frames compared to the default of 4 to account for more rapid photobleaching of mCherry compared to mYPet, resulted in >200 mCherry-Rep foci across N=77 cells, with 45±5% of these colocalized to mYpet-DnaB (note, using the default foci detection criteria resulted in only 11 Rep-mCherry foci tracks detected from these 77 cells, compared to >200 DnaB-mYPet foci, however, of these the proportion that were colocalized with mYpet-DnaB foci was still measured as ~45%).

Overexpression and Purification of mGFP-Rep and mGFP-RepC4ala

mGFP-rep and *mGFP-repC4ala* were sub-cloned from pAS79 and pAS124 respectively using XhoI and BamHI before ligation into pET14b cut similarly, creating pJLH237 and pJLH238 encoding histidine-tagged mGFP-Rep and histidine-tagged mGFP-RepC4ala respectively. pJLH237 and 238 were used to overexpress the mGFP-Rep fusions in HB222. Growth was carried out in F-Medium (28) at 37°C until OD_{600} ~ 0.7, overexpression was induced by the addition of 0.2% arabinose (w/v) and 1mM IPTG for 3 hours at 20°C. Cells were pelleted by centrifugation at 5000xg for 20 minutes at 4°C before flash freezing in 50 mM Tris-HCl pH 7.5, 10% sucrose (w/v) and storage at -80°C. Cell pellets were thawed on ice and the following additions were made (final concentrations indicated) 50 mM Tris-Cl pH 8.4, 20 mM EDTA pH 8.0, 150 mM KCl and 0.2 mg ml^{-1} lysozyme. After 10 min incubation on ice, Brij-58 was added to 0.1% (v/v of final concentration) with a further 20 min incubation on ice. The mixture was clarified by centrifugation at 148,000xg for 1 hour at 4°C and the supernatant recovered. DNA was precipitated from the resultant supernatant by dropwise addition of Polymin P to 0.075% (v/v) with stirring at 4°C for 10 minutes. The supernatant was recovered by centrifugation (30,000xg, 4°C for 20 minutes) before solid ammonium sulfate was added to 50% saturation whilst stirring at 4°C for 10 minutes. The pellet was recovered by centrifugation at 30,000xg at 4°C for 20 minutes and stored on ice overnight at 4°C. The protein pellet was then diluted in 20 mM Tris-HCl pH 7.9 and 5 mM imidazole until the conductivity matched that of 20 mM Tris-HCl pH 7.9 and 500 mM NaCl (buffer A) plus 5 mM imidazole. The Rep fusion proteins were purified by chromatography on a 1 ml His-trap FF crude column (GE healthcare) using a 20 ml wash with buffer A + 20 mM imidazole and a 20 ml gradient 20 mM to 1 M imidazole in buffer A, collecting 0.25 ml fractions. Peak fractions (~120 mM imidazole) were collected, and a Vivaspin 20 concentrator (100 kDa MWCO) (Sartorius)

was used to assess for concentration levels and for buffer exchange into 20 mM Tris-HCl pH 8.0, 500 mM NaCl, 1 mM EDTA, 1 mM DTT, 30% glycerol (v/v). Samples were then aliquoted and flash frozen in liquid nitrogen before storage at -80°C. Protein concentrations were determined by Bradford's assay.

Helicase assay

Unwinding of streptavidin-bound forks was assayed using a substrate made by annealing oligonucleotides oPM187B20 and oPM188B34. Reactions were performed in final volumes of 10 µL in 50 mM HEPES (pH 8); 10 mM DTT; 10 mM magnesium acetate; 2 mM ATP; 0.1 mg ml^{-1} BSA and 1 nM forked DNA substrate. Reactions were carried out as described in (18). Briefly, the reaction mixture was pre-incubated at 37°C for five minutes +/- 1 µM streptavidin (Sigma-Aldrich), then histidine-tagged helicase (as indicated) and biotin (Sigma-Aldrich) to 100 µM (acting as a trap for free Streptavidin) were added and incubation continued at 37°C for 10 minutes. Reactions were stopped with 2.5 µl of 2.5% SDS, 200 mM EDTA and 10 mg ml^{-1} of proteinase K. Reactions were then analysed by non-denaturing gel electrophoresis on 10% polyacrylamide TBE gels. The quantification of the unwinding and displacement of streptavidin from the fork was carried out as described (17).

Supplementary References

1. Baba, T., Ara, T., Hasegawa, M., Takai, Y., Okumura, Y., Baba, M., Datsenko, K.A., Tomita, M., Wanner, B.L. and Mori, H. (2006) Construction of *Escherichia coli* K-12 in-frame, single-gene knockout mutants: the Keio collection. *Mol. Syst. Biol.*, **2**, 2006 0008.
2. Atkinson, J., Gupta, M.K. and McGlynn, P. (2011) Interaction of Rep and DnaB on DNA. *Nucleic Acids Res.*, **39**, 1351-1359.
3. Bachmann, B.J. (1996) In Neidhardt, F. C., Curtiss III, R., Ingraham, J. L., Lin, E. C. C., Low, K. B., Magasanik, B., Reznikoff, W. S., Riley, M., Schaechter, M. and Umbarger, H. E. (eds.), *Escherichia coli and Salmonella cellular and molecular biology*. Second ed. ASM Press, Washington, DC, pp. 2460-2488.
4. Bernhardt, T.G. and de Boer, P.A. (2004) Screening for synthetic lethal mutants in Escherichia coli and identification of EnvC (YibP) as a periplasmic septal ring factor with murein hydrolase activity. *Molecular microbiology*, **52**, 1255-1269.

5. Guy, C.P., Atkinson, J., Gupta, M.K., Mahdi, A.A., Gwynn, E.J., Rudolph, C.J., Moon, P.B., van Knippenberg, I.C., Cadman, C.J., Dillingham, M.S. *et al.* (2009) Rep Provides a Second Motor at the Replisome to Promote Duplication of Protein-Bound DNA. *Mol. Cell*, **36**, 654-666.
6. Gupta, M.K., Guy, C.P., Yeeles, J.T., Atkinson, J., Bell, H., Lloyd, R.G., Marians, K.J. and McGlynn, P. (2013) Protein-DNA complexes are the primary sources of replication fork pausing in *Escherichia coli*. *Proc. Natl. Acad. Sci. U S A*, **110**, 7252-7257.
7. Mahdi, A.A., Briggs, G.S. and Lloyd, R.G. (2012) Modulation of DNA damage tolerance in *Escherichia coli recG* and *ruv* strains by mutations affecting PriB, the ribosome and RNA polymerase. *Mol. Microbiol.*, **86**, 675-691.
8. Rudolph, C.J., Upton, A.L., Stockum, A., Nieduszynski, C.A. and Lloyd, R.G. (2013) Avoiding chromosome pathology when replication forks collide. *Nature*, **500**, 608-611.
9. Beattie, T.R., Kapadia, N., Nicolas, E., Uphoff, S., Wollman, A.J., Leake, M.C. and Reyes-Lamothe, R. (2017) Frequent exchange of the DNA polymerase during bacterial chromosome replication. *eLife*, **6**.
10. Datsenko, K.A. and Wanner, B.L. (2000) One-step inactivation of chromosomal genes in *Escherichia coli* K-12 using PCR products. *Proc. Natl. Acad. Sci. U S A*, **97**, 6640-6645.
11. Landgraf, D., Okumus, B., Chien, P., Baker, T.A. and Paulsson, J. (2012) Segregation of molecules at cell division reveals native protein localization. *Nat. Methods*, **9**, 480-482.
12. Boubakri, H., de Septenville, A.L., Viguera, E. and Michel, B. (2010) The helicases DinG, Rep and UvrD cooperate to promote replication across transcription units *in vivo*. *EMBO J.*, **29**.
13. Myka, K.K., Hawkins, M., Syeda, A.H., Gupta, M.K., Meharg, C., Dillingham, M.S., Savery, N.J., Lloyd, R.G. and McGlynn, P. (2017) Inhibiting translation elongation can aid genome duplication in *Escherichia coli*. *Nucleic Acids Res.*, **45**, 2571-2584.
14. Sandler, S.J., Marians, K.J., Zavitz, K.H., Coutu, J., Parent, M.A. and Clark, A.J. (1999) *dnaC* mutations suppress defects in DNA replication- and recombination- associated functions in *priB* and *priC* double mutants in *Escherichia coli* K-12. *Mol. Microbiol.*, **34**, 91-101.
15. Mahdi, A.A., Buckman, C., Harris, L. and Lloyd, R.G. (2006) Rep and PriA helicase activities prevent RecA from provoking unnecessary recombination during replication fork repair. *Genes Dev.*, **20**, 2135-2147.
16. Atkinson, J., Gupta, M.K., Rudolph, C.J., Bell, H., Lloyd, R.G. and McGlynn, P. (2011) Localization of an accessory helicase at the replisome is critical in sustaining efficient genome duplication. *Nucleic Acids Res.*, **39**, 949-957.
17. Bruning, J.G., Howard, J.A. and McGlynn, P. (2016) Use of streptavidin bound to biotinylated DNA structures as model substrates for analysis of nucleoprotein complex disruption by helicases. *Methods*, **108**, 48-55.
18. Bruning, J.G., Howard, J.A.L., Myka, K.K., Dillingham, M.S. and McGlynn, P. (2018) The 2B subdomain of Rep helicase links translocation along DNA with protein displacement. *Nucleic Acids research*.
19. Wollman, A.J., Shashkova, S., Hedlund, E.G., Friemann, R., Hohmann, S. and Leake, M.C. (2017) Transcription factor clusters regulate genes in eukaryotic cells. *eLife*, **6**.

20. Wollman, A.J., Syeda, A.H., McGlynn, P. and Leake, M.C. (2016) Single-Molecule Observation of DNA Replication Repair Pathways in *E. coli*. *Adv. Exp. Med. Biol.*, **915**, 5-16.
21. Miller, H., Zhou, Z., Wollman, A.J. and Leake, M.C. (2015) Superresolution imaging of single DNA molecules using stochastic photoblinking of minor groove and intercalating dyes. *Methods*, **88**, 81-88.
22. Thompson, R.E., Larson, D.R. and Webb, W.W. (2002) Precise nanometer localization analysis for individual fluorescent probes. *Biophysical journal*, **82**, 2775-2783.
23. Reyes-Lamothe, R., Sherratt, D.J. and Leake, M.C. (2010) Stoichiometry and architecture of active DNA replication machinery in *Escherichia coli*. *Science*, **328**, 498-501.
24. Llorente-Garcia, I., Lenn, T., Erhardt, H., Harriman, O.L., Liu, L.N., Robson, A., Chiu, S.W., Matthews, S., Willis, N.J., Bray, C.D. *et al.* (2014) Single-molecule *in vivo* imaging of bacterial respiratory complexes indicates delocalized oxidative phosphorylation. *Biochim. Biophys. Acta*, **1837**, 811-824.
25. Wollman, A.J. and Leake, M.C. (2015) Millisecond single-molecule localization microscopy combined with convolution analysis and automated image segmentation to determine protein concentrations in complexly structured, functional cells, one cell at a time. *Faraday discussions*, **184**, 401-424.
26. Leake, M.C., Chandler, J.H., Wadhams, G.H., Bai, F., Berry, R.M. and Armitage, J.P. (2006) Stoichiometry and turnover in single, functioning membrane protein complexes. *Nature*, **443**, 355-358.
27. Bakshi, S., Siryaporn, A., Goulian, M. and Weisshaar, J.C. (2012) Superresolution imaging of ribosomes and RNA polymerase in live *Escherichia coli* cells. *Mol. Microbiol.*, **85**, 21-38.
28. Dallmann, H.G., Thimmig, R.L. and McHenry, C.S. (1995) DnaX complex of Escherichia coli DNA polymerase III holoenzyme. Central role of tau in initiation complex assembly and in determining the functional asymmetry of holoenzyme. *J. Biol. Chem.*, **270**, 29555-29562.
